# Downregulation of Ubiquitin-Specific Protease 15 (USP15) Does Not Provide Therapeutic Benefit in Experimental Mesial Temporal Lobe Epilepsy

**DOI:** 10.1007/s12035-023-03692-2

**Published:** 2023-10-24

**Authors:** Ute Häussler, João Neres, Catherine Vandenplas, Caroline Eykens, Irena Kadiu, Carolin Schramm, Renaud Fleurance, Phil Stanley, Patrice Godard, Laurane de Mot, Jonathan van Eyll, Klaus-Peter Knobeloch, Carola A. Haas, Stefanie Dedeurwaerdere

**Affiliations:** 1 Experimental Epilepsy Research, Department of Neurosurgery, Medical Center - University of Freiburg, Faculty of Medicine, University of Freiburg, Breisacher Strasse 64, 79106 Freiburg, Germany; 2 BrainLinks-BrainTools Center, University of Freiburg, Georges-Koehler-Allee 201, 79110 Freiburg, Germany; 3 Early Solutions, UCB Biopharma SRL, Chemin du Foriest, 1420 Braine L’Alleud, Belgium; 4 Early Development Statistics, UCB Celltech, 208 Bath Road, Slough, Berkshire, SL1 3WE UK; 5 Institute for Neuropathology, Medical Center - University of Freiburg, Faculty of Medicine, University of Freiburg, Breisacher Strasse 64, 79106 Freiburg, Germany; 6 CIBSS – Centre for Integrative Biological Signalling Studies, Freiburg, Germany; 7 Center for Basics in NeuroModulation, Faculty of Medicine, University of Freiburg, 79106 Freiburg, Germany; 8 Bernstein Center Freiburg, University of Freiburg, Hansastr. 9a, 79104 Freiburg, Germany

**Keywords:** Neuroinflammation, TGF-β, IFN-α/β, NRF2, Microglia, Seizure, Kainate

## Abstract

Structural epilepsies display complex immune activation signatures. However, it is unclear which neuroinflammatory pathways drive pathobiology. Transcriptome studies of brain resections from mesial temporal lobe epilepsy (mTLE) patients revealed a dysregulation of transforming growth factor β, interferon α/β, and nuclear factor erythroid 2-related factor 2 pathways. Since these pathways are regulated by ubiquitin-specific proteases (USP), in particular USP15, we hypothesized that USP15 blockade may provide therapeutic relief in treatment-resistant epilepsies. For validation, transgenic mice which either constitutively or inducibly lack Usp15 gene expression underwent intrahippocampal kainate injections to induce mTLE. We show that the severity of *status epilepticus* is unaltered in mice constitutively lacking Usp15 compared to wild types. Cell death, reactive gliosis, and changes in the inflammatory transcriptome were pronounced at 4 days after kainate injection. However, these brain inflammation signatures did not differ between genotypes. Likewise, induced deletion of Usp15 in chronic epilepsy did not affect seizure generation, cell death, gliosis, or the transcriptome. Concordantly, siRNA-mediated knockdown of Usp15 in a microglial cell line did not impact inflammatory responses in the form of cytokine release. Our data show that a lack of USP15 is insufficient to modulate the expression of relevant neuroinflammatory pathways in an mTLE mouse model and do not support targeting USP15 as a therapeutic approach for pharmacoresistant epilepsy.

**Supplementary Information:**

The online version contains supplementary material available at 10.1007/s12035-023-03692-2.

## Introduction

Epilepsy is one of the most common neurologic disorders that affect over 50 million people worldwide. It is characterized by an enduring predisposition to generate seizures and can be associated with significant comorbidities such as cognitive and emotional dysfunction and increased mortality [1, 2]. Despite the diversity of epilepsy syndromes and their underlying causes [3, 4], significant progress has been made during the past decades with anti-seizure medication (ASM) that is able to control seizures in about 70–80% of the patients (reviewed in [5]). For certain etiologies, drug resistance is particularly high and for mesial temporal lobe epilepsy (mTLE) with hippocampal sclerosis reports indicate that only around 30% of patients are seizure-free with current medication [6–9]. Available ASMs are symptomatic and do not address the underlying causes or progression of epilepsy. It is therefore crucial to develop new therapeutic strategies for pharmacoresistant epilepsy patients [5, 10].

Neuroinflammatory pathways have emerged as key elements in the development and progression of epilepsy, representing potential targets to develop novel disease-modifying therapies [11–15]. In surgically resected tissue from patients with mTLE, markers of neuroinflammation including microglia activation, reactive astrogliosis, peripheral immune cell infiltration, and expression of soluble mediators of inflammation (cytokines, chemokines) and damage-associated molecular patterns (DAMPs) are upregulated [16–18]. A similar proinflammatory response was observed in rodent models of mTLE [16, 17, 19].

The ubiquitin–proteasome system regulates protein function and important physiological processes through reversible linkage of ubiquitin (ubiquitination) to target proteins. Ubiquitination may mark a protein for degradation or regulate its function in general. Like most posttranslational modifications, ubiquitination is a reversible process counteracted by ubiquitin-specific proteases (deubiquitinases (DUBs)). As druggable molecules, DUBs have emerged as promising targets for drug discovery in several diseases such as cancer, neurodegeneration, immunity, and inflammation [20–22]. A recent study identified the DUB USP15 as a key regulator in the pathogenesis of neuroinflammation in experimental cerebral malaria and experimental autoimmune encephalomyelitis [23]. Within that context, a single-point mutation in the Usp15 gene or its full knockout (KO) had a remarkable protective effect by reducing neuroinflammation and increasing survival, most likely by dampening the type I interferon (IFN) response [23]. In addition, cell-based experiments provide evidence that USP15-dependent deubiquitination activates transforming growth factor (TGF)-β signaling via stabilization of the type I TGF-β receptor (TGF-βR1) directly as well as via binding to the SMAD7-SMAD-specific E3 ubiquitin protein ligase 2 (SMURF2) and regulating receptor-activated SMAD-dependent transcription [24–26]. Furthermore, USP15 was reported to regulate the nuclear factor erythroid 2-related factor 2 (NRF2)-pathway by stabilizing NRF2 via deubiquitination of KEAP1, which plays a protective role against oxidative stress [27].

Although the role of USP15 in chronic epilepsy is unknown, central roles of its downstream pathways have been established: Transgenic mice overexpressing IFN-α in astrocytes have a progressive phenotype with severe seizures [28]. Nrf2-deficient mice are more vulnerable to kainic acid (KA)–induced seizures [29], whereas the constitutive upregulation of NRF2 resulted in less generalized seizures, increased neuroprotection, and normalized astroglial activation [30]. TGF-β, its receptors, and R-SMADS are upregulated in mTLE patients with hippocampal sclerosis [31]. TGF-β upregulation in mTLE animal models is involved in epileptogenesis and seizure generation as reduced blood–brain barrier integrity causes the leaking of albumin which binds to the TGF-β receptor [32]. This could be prevented by the application of TGF-β pathway blockers [33, 34].

**Fig. 1:**
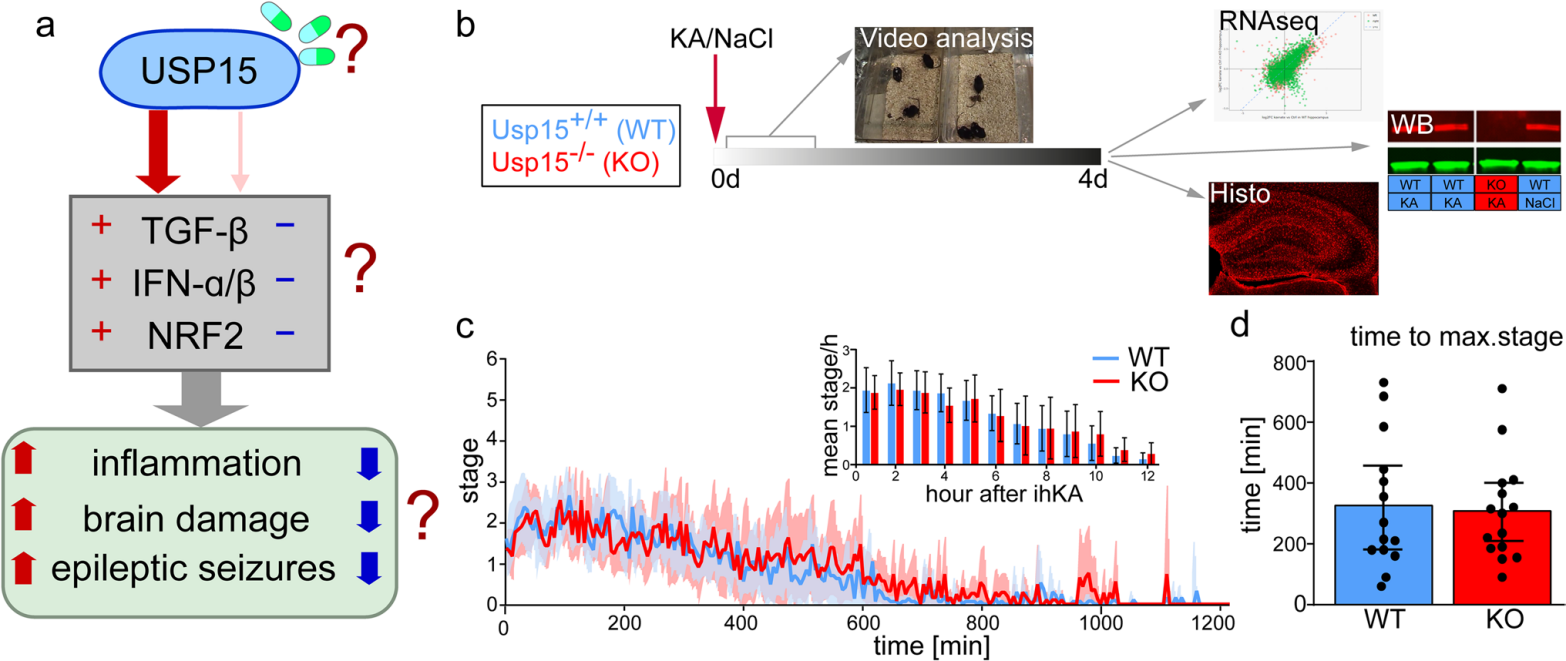
*Status epilepticus* is comparable in Usp15^−/−^ mice and wild-type littermates. **a** Initial hypothesis for USP15 action in mTLE. Left side: USP15 activates/stabilizes important pathways involved in inflammation, brain damage, and ultimately seizure occurrence in mTLE. Right side: As a druggable molecule, the suppression of USP15 might result in lower activation of pathways involved in inflammation and ultimately in reduced pathophysiology in mTLE. **b** Schematic depicting the experimental design of induction of *status epilepticus* in Usp15^−/−^ (KO) and wild-type (WT) mice compared with video monitoring, followed by RNAseq, Western blot (WB), and histological analysis. **c** Mean time course of the maximum stage during *status epilepticus* reached within 5-min-long windows aligned to the time point of awaking from anesthesia. WT mice (*n* = 14) are shown in blue, Usp15^−/−^ mice (KO, *n* = 15) are shown in red, shadows in corresponding color depict 95% confidence intervals (CI). The time courses are overlapping. Inset: Area under the curve per hour for the first 12 h after ihKA injection (mean ± 95% CI) did not differ between WT and USP15^−/−^ mice (multiple *t* tests with Welch’s correction not significant (ns)). **d** Maximum stage reached during *status epilepticus* (mean ± 95% CI, individual values) did not differ between Usp15^−/−^ and WT littermates (Student’s *t* test ns)

Using the pilocarpine mTLE animal model, we have performed a transcriptome study, which identified gene networks with differential co-expression. These gene networks were highly conserved in human tissue and regulated under the control of the pathways mentioned above [35]. In addition, pilot data in the intrahippocampal KA (ihKA) model in mice indicated clear alterations in the type I IFN response as well as NRF2 and TGF-β pathways. Given the modulating role of USP15 in all three pathways, it is reasonable to hypothesize that the inhibition of USP15 could lead to an effective dampening of excessive neuroinflammation in mTLE, finally leading to improvement of the pathobiology and seizures (Fig. 1a).

To investigate this hypothesis, we used constitutive and inducible KO mouse lines and analyzed the impact of deletion of the Usp15 gene on epilepsy-dependent histological changes and downstream modulation of IFN-α/β, TGF-β, and NRF2 pathways acutely after *status epilepticus* and determined whether this has disease-modifying effects in chronic epilepsy.

## Methods

### Animals

Experiments were carried out with mice either constitutively lacking Usp15 expression (Usp15^−/−^) or allowing its conditional inactivation via the Cre/loxP system (Usp15^fl/fl^CAGCreERT2^+^). To generate these mice, Exon 8, encompassing the codon for the active site cysteine, which is essential for protease activity, was flanked by loxP sites employing homologous recombination in murine embryonic stem cells (E14.1). The targeting vector contained 5′ and 3′ homologous regions, a frt-flanked pgk-neomycine resistance gene, and two loxP sites, which upon homologous recombination were inserted upstream and downstream of exon 8. Successful 5′ and 3′ integration was validated by Southern blot analysis using probes located upstream and downstream of homology regions. ES cells harboring the targeted allele were used to generate germline chimeras, which were subsequently mated to a FLP deleter strain [36] to eliminate the FRT flanked neo resistance gene. Resulting Usp15^fl/+^ mice were crossed to the CAGCre-ER™ strain [37] to generate the Usp15^fl/fl^CAGCreERT2^+^ line employed for inducible inactivation by tamoxifen injection. Alternatively, Usp15^−/−^ mice were generated by mating Usp15^fl/+^ mice to germline-expressing CMVCre animals [38]. Respective wild-type (WT) littermates of the constitutive KO line (i.e., Usp15^+/+^ mice) will be referred to as WT in the following. In the Usp15^fl/fl^CAGCreERT2^+^ line, inducible KO mice carrying two floxed Usp15 alleles and one allele of Cre recombinase will be referred to as Usp15^fl/fl^Cre^+^ before tamoxifen treatment and Usp15^Δ/Δ^ after tamoxifen treatment. The respective Cre-negative littermates will be referred to as Usp15^fl/fl^.

The genotype of Usp15^−/−^ mice and the level of USP15 protein deletion in the brain of the Usp15^Δ/Δ^ following tamoxifen treatment were confirmed at the end of the in vivo experiments by Western blot (Suppl. Figure 1).

Before and during experiments, mice were kept in a 12-h light/dark cycle at room temperature (RT, 22 ± 1 °C) with food and water ad libitum. Animal procedures were carried out in accordance with the European Community’s Council Directive (2010/63/EU) and approved by the regional council (Regierungspräsidium Freiburg). In total, 123 mice were used for the study.

### In Vivo Studies

#### Surgery, Data Acquisition, and Analysis

##### Constitutive Usp15 Knockout

Surgery: Unilateral, ihKA injections were performed in adult male Usp15^−/−^ and WT littermates (study protocol Fig. 1b). Mice were anesthetized and received analgesic treatment (100 mg/kg ketamine (CEVA animal health, FR), 5 mg/kg xylazine (Rompun®, Bayer, DE), 0.1 mg/kg atropine (Braun, DE), i.p. and 4 mg/kg carprofen (Carprieve®, CP-Pharma, DE), s.c.), and placed into a stereotaxic frame (David Kopf Instruments, CA, USA) in flat skull position. KA solution (50 nl, 20 mM in 0.9% sterile NaCl, KA #487–79-6, Tocris Bioscience, UK) was injected into the right dorsal hippocampus (coordinates from bregma (in mm): anterioposterior (AP) =  − 2.0, mediolateral (ML) =  − 1.4, dorsoventral (DV) =  − 1.9) with a metal cannula (Harry Rieck Edelstahl, DE) connected to a Hamilton syringe (Hamilton Bonaduz AG, CH) and a micropump, as described before [39]. Controls received NaCl solution only. After injection, mice were transferred to their home cages and kept under video surveillance for at least 8 h (mean 18.1 h, range 8–23 h). Mice were then kept in groups in their home cages and perfused at 4 days after KA injection.

Data acquisition: Video analysis of *status epilepticus* was performed by an observer blinded to the genotype. First, the time point when mice awakened from anesthesia was determined. The behavior was then classified according to a modified Racine staging scheme [40]. The scheme was adapted to the characteristic behavior of mice with unilateral ihKA injections: 0 — awake immobility/rest or active behavior (running, grooming, feeding etc.; this stage might comprise electrographic *status epilepticus*, in particular during rest/immobility [41], but no clear behavioral signs of *status epilepticus*), 1 — head nodding or facial automatisms, 2 — rotations, 3 — forelimb clonus, 4 — rearing, 5 — rearing and falling, 6 — jumping. Videos were split in consecutive 5-min time windows, and the highest stage reached within each time window was determined.

Data analysis: To compare *status epilepticus*, the area under the curve (AUC) for the time course of stages was calculated for each mouse for the first 12 h after ihKA, respectively, and compared with multiple Student’s *t* tests with Welch’s correction and Benjamini-Krieger-Yekutieli method to correct for the false discovery rate (FDR). Furthermore, the maximum stage reached, and the occurrence count (number of time windows with the particular stage as maximum) and the time to reach stages 3–6 were analyzed. A Shapiro–Wilk test for normal distribution was performed followed by either a Student’s *t* test or a Mann–Whitney test if values were not normally distributed.

Statistical analysis of the entire study was performed with GraphPad Prism (version 9.3.0, GraphPad Software Inc., CA, USA) or with R (version 4.0.2, The R Foundation, AT). Significance levels were set to *p < 0.05, **p < 0.01, ***p < 0.001, ****p < 0.0001. In the following, the statistical tests applied for each analysis will be described together with the respective data analyses.

##### Inducible Usp15 Deletion

Surgery: Usp15^fl/fl^Cre^+^ mice and Usp15^fl/fl^ littermates were injected with KA (or NaCl) as described above (study protocol in Fig. 4a). Seven days later, mice were implanted with electrodes (anesthesia; see above with 0.1 mg/kg buprenorphine added, Temgesic®, Indivior, UK). Custom-made platinum-iridium wire electrodes (Ø125 µm, Teflon-insulated, World Precision Instruments, FL, USA) were implanted in both hippocampi (coordinates from bregma in mm: AP − 2.0, ML − 1.4/ + 1.4, DV − 1.9) and two jeweler’s screws in the skull anterior to bregma served as ground and reference, as described previously [41]. Wires were soldered to a connector permanently mounted on the skull with cyanoacrylate (Pattex, DE) and dental cement (Paladur, Kulzer, DE). Mice received buprenorphine and carprofen analgesia for the days after surgery.

Data acquisition: The local field potential (LFP) was recorded from day 14 to day 18. This was followed by tamoxifen treatment for 5 days (day 21–25; Sigma-Aldrich/Merck, DE, 0.15 ml of a 10 mg/ml solution in corn oil, Sigma-Aldrich/Merck) to delete Usp15 expression. Subsequent LFP recordings were performed daily for day 28–32, day 35–39, and day 42–46. Four mice were recorded only for 39 instead of 46 days; another four mice were recorded for 45 days. To record, mice were connected via a cabled miniature preamplifier (MPA 8i, Multichannel Systems/SmartEphys, DE) and were allowed to freely move in individual recording cages. Following customization to the recording setup for 1 h, mice were recorded for 2 h in the afternoon. Signals were amplified (1000-fold, PGA32, Multichannel Systems/SmartEphys), bandpass filtered (1 Hz–5 kHz), and digitized (sampling rate 20 kHz; Power1401 with Spike2 software, CED, UK). NaCl-injected mice were not recorded (except for test recordings to verify that they are not epileptic) but implanted, weighted, handled daily, and tamoxifen-injected to guarantee equal treatment otherwise. Usp15^fl/fl^Cre^+^ and Usp15^fl/fl^ mice were mixed in every experiment series.

Data analysis: Epileptic spikes were analyzed with a custom-made algorithm described previously [42]. In brief, after manually removing artifacts, epileptic population spikes were detected based on a combination of amplitude and frequency composition. Next, clustering of spikes into bursts of different severity (low, medium, and high spike load) was performed according to spike numbers, median inter-spike-intervals, and standard deviation (SD) of inter-spike intervals using self-organizing maps [42]. We then calculated the EA ratio (summed duration of epileptic activity/total recording duration) and the weekly EA ratios for 1–3 weeks after tamoxifen were analyzed separately using analysis of covariance (ANCOVA) with the pre-tamoxifen week EA ratio as the covariate. The cumulative distribution of the durations of high spike-load events was analyzed separately for each week using a logistic regression assuming proportional odds. In addition, weekly averages for severe, moderate, and mild bursts were calculated with an ANCOVA with the pre-tamoxifen values as the covariate.

### Perfusion and Preparation of Brains

Perfusion was performed at day 4 for Usp15^−/−^ and WT mice and on the day of the last recording for Usp15^Δ/Δ^ and Usp15^fl/fl^ mice. If brains were used for transcriptome analysis, mice were anesthetized with ketamine (100 mg/kg) and medetomidine (Dormitor, Provet AG, CH; 1 mg/kg), and transcardial perfusion was performed with 30-ml phosphate-buffered saline (PBS, pH 7.4). Brains were excised immediately afterwards, and both hippocampi and cortices, respectively, were prepared in ice-cold PBS, snap frozen in individual RNAse-free, sterile tubes in liquid nitrogen and stored at − 80 °C until all samples were collected. If brains were used for histology, mice were anesthetized (250 mg/kg ketamine, 12.5 mg/kg xylazine, 0.2 mg/kg acetylpromazine) and perfused with 0.9% NaCl solution for 1 min, followed by 4% paraformaldehyde (PFA, Carl Roth GmbH, DE) for ~ 4 min. Brains were excised and stored in PFA for 4 h, transferred in phosphate buffer and processed the next day.

### Histological Analysis

#### Histopathology Evaluation

Heart, lungs, kidneys, liver/gallbladder, thymus, adrenals, mesenteric lymph node, skeletal muscle, spleen, sternum, and bone marrow were collected from 7 Usp15^−/−^ females, 7 matching WT mice, 8 Usp15^Δ/Δ^ mice, and 10 control mice (given tamoxifen only). In addition, forestomach/stomach, duodenum, jejunum, ileum with Peyer’s patches, cecum, colon, rectum, brain, axillary lymph node, and femur were also sampled from the Usp15^Δ/Δ^ mice and Usp15^fl/fl^ mice. The selected tissues were fixed in 10% neutral buffered formalin, trimmed, embedded in paraffin, processed to 4-µm-thick histological slides, and stained with hematoxylin and eosin (#3801654, HistoCore SPECTRA H&E STAIN SYSTEM S1) on a HistoCore Spectra workstation (both Leica Biosystems, DE). The microscopic evaluation was conducted by a board-certified veterinary pathologist.

#### Immunocytochemistry

For immunostaining, 50-µm-thick horizontal sections were sliced on a vibratome (VT100S, Leica, DE) in series of five and washed in phosphate buffer. Preincubation was performed with 1% bovine serum albumin (BSA) and 0.25% Triton X-100 (both Sigma-Aldrich/Merck) in PB for 30 min. The following primary antibodies were applied (with 1% BSA and 0.1% Triton in PB; 4 h at RT + overnight at 4 °C): Neuronal nuclei antigen (NeuN, guinea pig polyclonal, #266044, 1:1000, Synaptic Systems, DE), ionized calcium-binding adapter molecule 1 (Iba1, rabbit polyclonal, #019–19741, 1:500, WAKO Pure Chemical Cooperation, JP), glial-fibrillary acidic protein (GFAP, rabbit polyclonal, Z0334, 1:500, Dako, CA, USA), or CD68 (rabbit polyclonal, ab125212, 1:500, Abcam, UK). After washing in PB, incubation with a secondary antibody (Cy™ 5-coupled 1:100, Cy™ 3-coupled 1:200, Dako) was performed together with 4′,6-diamidino-2-phenylindole counterstaining (DAPI, 1:10,000, Roche Diagnostics, DE) for 2 h at RT. Sections were mounted on microscope slides and embedded with Immumount (Thermo Scientific, DE, USA).

#### Microscopy and Image Analysis

Images were taken with a Zeiss AxioImager 2 with Zen software (Carl Zeiss AG, DE) with a tenfold objective with identical exposure times for each antibody, respectively. For image analysis, an observer blinded to the genotype (blinding towards KA or NaCl treatment is impossible due to the salient changes caused by KA injection) selected sections within a range from − 1.58 to − 2.46 mm behind bregma according to the mouse brain atlas [43]. Only sections without major damage were used for analysis resulting in ~ 4 to 6 slices/mouse/antibody. Fluorescence intensity measurements were carried out using ImageJ (v1.53a, NIH, USA).

Granule cell layer width was measured in the upper blade at five positions (at least 250 µm away from the apex) and averaged in NeuN-labeled sections. To analyze the density of NeuN expression in CA3 and CA1, the images were converted to 16-bit grey-scale, and the mean grey values in both hippocampi were measured. A threshold was set to mean + 1 SD and pixels with grey values > threshold were determined. A rectangular region of interest (300 × 200 µm for CA3, 400 × 200 µm for CA1) was placed above the pyramidal cell layer of either region and the relative area of pixels with grey values > threshold was measured in the ipsi- and contralateral hippocampus. The results were compared with unpaired Student’s *t* tests for the constitutive Usp15 KO experiment series and a two-way analysis of variance (ANOVA) with Tukey post hoc test for the inducible Usp15 deletion experiment series.

To measure density of GFAP, Iba1, and CD68 expression, images were converted to 16-bit grey-scale. The background staining of the respective section was determined in a 500 × 500-µm square in the left cortex above the hippocampus (in an area without any electrode traces) and subtracted from the entire image. The integrated density was then measured for the whole ipsi- and contralateral hippocampus for all genotypes and treatments, respectively. The integrated density function of ImageJ corresponds to mean grey value × area in mm^2^. Integrated density values were log-transformed prior to statistical analysis to induce symmetry and equal variance to adhere to the assumption of normality. For the constitutive KO experiment series, unpaired Student’s *t* tests were performed assuming equal variance on the log scale. Results were back-transformed to the original scale, and geometric means are displayed. For the inducible deletion series, analyses were performed using a two-way ANOVA with Tukey’s multiple comparison test on the log-transformed data for the different comparisons followed by transformation to the original scale to display the geometric means.

### Transcriptome Analysis

#### RNA Isolation

Total RNA was extracted from both left and right mouse hippocampi using RNeasy Plus Mini Kit (Qiagen, 74,134, NL) following manufacturer’s instructions. RNA concentration was measured with a NanoDrop ND-1000 Spectrophotometer (Thermo Scientific). RNA quality was assessed using a Bio-Rad Experion Automated Electrophoresis System (RQI ≥ 7.3; Bio-Rad Laboratories, UK).

#### Library Preparation, RNA Sequencing, and Differential Expression Analysis

RNA-seq libraries were prepared using the NEBNext Ultra II Directional RNA Library Prep Kit (Illumina®, NEB #E7760S/L, New England BioLabs Inc, MA, USA), following the manufacturer’s guidelines and sequenced with 151 nucleotides per read. Reads were filtered for quality and contamination using bbduk from the BBTools suite (version 38.31, https://jgi.doe.gov/data-and-tools/software-tools/bbtools/). Specifically, we filtered out common adapters, bacterial sequences, PhiX, ribosomal sequences, reads with Phred score ≥ 20 in at least one end, reads < 31 nucleotides, reads with low complexity, and homopolymer reads. On average, 77% (± 1%) of the fragments were kept for downstream analysis.

Filtered fragments were mapped on mouse GRCm38 reference transcriptome downloaded from Ensembl ftp (Ensembl project, UK; https://www.ensembl.org/info/data/ftp/index.html). The Salmon quasi-mapping-based mode (version 0.11.3 [44], https://salmon.readthedocs.io/en/nb/index.html) was used for quantifying transcripts, with default k-mer size of 31 used to construct the reference transcriptome index. On average, 94% (± 1%) of the processed fragments were correctly mapped on the reference transcriptome.

Transcript counts were summarized to gene level and additionally scaled using library size and average transcript length over samples [45]. We only kept genes with counts above 10 in at least 10 samples (in total 15,877 of 35,880 genes). Gene counts were finally normalized by applying the TMM method (weighted trimmed mean of M-values) [46] and log2-transformed.

Major batch effects were ruled out using several methods, including running principal component analysis (PCA) on normalized gene values and assessing association between each PC and possible covariates (using ANOVA for categorical covariate and Spearman’s correlation for numerical covariate). Differential expression analysis was used to assess the effect of genotype (comparing cohorts) and injection type (comparing left NaCl and right KA hippocampi) on RNA-seq data. For this analysis, TMM-normalized gene counts were quantile-normalized and log2-transformed using voom ([47], https://rdrr.io/bioc/limma/man/voom.html), then the significance of the gene differential expression was assessed using limma ([48], https://rdrr.io/bioc/limma/). Next, the response was assessed through functional enrichment analysis using GSEA (fgsea R package, [49], https://rdrr.io/bioc/fgsea/) on gene lists pre-ranked by logFC for each comparison.

#### Western Blot

Western blot analysis was only performed for mice that underwent transcriptomic analyses, as brains used for histology had to be intact. One brain hemisphere (without hippocampus) from ihKA/ihNaCl mice was placed in a Precellys tube containing ceramic beads (CK14_7ml, Bertin Technologies, FR) and 2 × concentrated Cell Lysis Buffer (approx. 10 × the volume of the tissue; #9803, Cell Signaling, NL) containing 2 × concentrated Protease/Phosphatase Inhibitor Cocktail (#5872, Cell Signaling). Homogenization was performed in the Precellys tube (2 × 20-s pulses at 5000 RPM). The homogenates were centrifuged at 4 °C at 16,000 RPM, and the pellets discarded. The total protein concentration of the cleared homogenates was determined using the Pierce™ BCA assay kit (#23227, ThermoFisher Scientific). Proteins were separated by SDS-PAGE (20 μg protein load) on 4–15% Criterion™ TGX™ precast midi protein gels (#5671084, Bio-Rad), followed by transfer to nitrocellulose membranes (#1704159, Bio-Rad) using a Trans-Blot Turbo transfer system (Bio-Rad). The membranes were blocked for 2 h at 4 °C with Intercept® (TBS) Blocking Buffer (#927–60001, Li-cor Biosciences, DE) and then incubated with the primary antibody solution (antibody list in Suppl. Table 1) for ~ 10–14 h at 4 °C. After washing 5 × with Tris-buffered saline with Tween20 (TBST), the membranes were incubated with secondary antibodies (IRDye® 680RD goat anti-mouse IgG — no. 926–68070 and IRDye 800CW goat anti-rabbit IgG — no. 926–32211, Li-cor Biosciences) in TBST for 2 h at 4 °C and then washed 5 × with TBST. Membrane imaging was performed on an Odyssey® CLx imaging system (Li-cor), and band quantification was performed using the Image Lab software (Bio-Rad). Values were measured relative to β-actin loading control and displayed as % of the respective treatment control. After a Shapiro–Wilk test to confirm normality, values were compared with a Student’s *t* test for pairwise comparison or one-way ANOVA for groups followed by Dunnett’s (comparison to control) or Tukey’s multiple comparison tests (all-to-all comparison).

### BV2 Mouse Microglial Cell Line Assays

#### Culturing and siRNA Transfection

The murine BV2 microglial cell line (Banca Cellule ICLC, IT) was grown in Dulbecco’s modified Eagle’s medium (DMEM) high glucose (#11965092), with GlutaMAX supplement (#35050038) and supplemented with 10% fetal bovine serum (FBS, #26140079) and 1% penicillin/streptomycin (P/S, #15070063) (all ThermoFisher Scientific, USA). Cells were maintained in a 5% CO_2_ humidified incubator at 37 °C and underwent at least two passages before usage in cell culture experiments. The passage number used across all experiments with the BV2 line was P28-33.

For siRNA transfection, BV2 cells were plated in either 24-well or 96-well culture plates at a density of 190 K and 32 K cells/well, respectively. The cells were transfected with 27.5 nM of the scrambled or Usp15 siRNA pools (D-001810–10-05 and L-040417–01-0010, Dharmacon, CO, USA) using Viromer® BLUE (Viromer in the following; Lipocalyx GmbH, DE) as transfection reagent for 48 h in DMEM supplemented with 10% FBS and 1% P/S. Knockdown efficiency was determined using Western blot for which cells were lysed, and the cleared lysates were analyzed as described above.

#### Cytokine Release, Phagocytosis, and Lysosomal Assay

At 48 h after siRNA transfection, BV2 cells were stimulated for 24 h with lipopolysaccharide (LPS, from *Escherichia coli* O55:B5, L6529-1MG, Sigma-Aldrich/Merck) at a concentration of 1 μg/ml. Supernatants from cells treated with either Viromer alone or with Viromer and the scrambled or Usp15 siRNA pool were collected in ultra-low attachment plates (Corning®Costar®, CLS7007-24EA, Sigma-Aldrich/Merck) and stored at − 80 °C until further use. Cytokine levels were measured using the MSD® V-Plex Proinflammatory Panel 1 mouse Kit (K15048D, Meso Scale Discovery, MD, USA) according to the manufacturer’s instructions. All cell culture fluids were used undiluted, except for TNFα measurements for which supernatants were diluted 1:10 v/v. Values were compared with a two-way ANOVA, followed by Dunnett’s multiple comparison relative to Viromer control to compare siRNA-mediated effects within the PBS/LPS treatment groups, respectively.

For the phagocytosis assays, pHrodo®-conjugated zymosan particles (P35364, ThermoFisher Scientific) were added 48 h after the siRNA transfection at a concentration of 2.5 µg/ml per well. Subsequently, time-lapse images were recorded using the Incucyte® S3 live cell imaging system (Sartorius, DE; 20 × , 4 images per well after zymosan treatment). Phagocytosis was assessed by measuring the integrated intensity per well and normalizing it to the cell confluence area. Phagocytic activity was represented as percentage of phagocytosis relative to the Viromer control and compared with a repeated-measures two-way ANOVA. Following acquisition, the cells were washed with PBS, fixed for 10 min in 4% PFA and blocked for 1 h at RT in PBS supplemented with 0.3% triton and 10% normal donkey serum (D9663, Sigma-Aldrich/Merck). BV2 cells were stained with DAPI (#62,248, ThermoFisher Scientific), and images were acquired using an Opera® Phenix screening system (Revvity Inc, MA, USA).

Lysosomal assays were performed 48 h after siRNA transfection. Cells were incubated with Bafilomycin (tlrl-baf1, InvivoGen, FR) at a final concentration of 100 nM in DMSO for 1 h at 37 °C. LysoSensor™ Green DND-189 and LysoTracker™ Red DND-99 (L7535 and L7528, both ThermoFisher Scientific) were then added to the culture to a final concentration of 1 µM and 100 nM, respectively. Following 5 min of incubation at 37 °C, the medium was changed, and time-lapse images were recorded with the Incucyte® S3 live cell imaging system (20 × , 4 images per well at indicated times after dye addition). The LysoSensor and LysoTracker signal were assessed by measuring the integrated intensity per well and normalizing it to the confluence area. The signal is represented as percentage relative to the Viromer control. Data was compared with a Kruskal–Wallis test with Dunn’s multiple comparison test.

#### PCR

The cells were washed with PBS for RNA extraction with the RNeasy Plus Mini kit (#74134, Qiagen), following the manufacturer’s instructions. cDNA was synthesized from 0.4 µg total RNA using Applied Biosystems™ High-Capacity cDNA Reverse Transcription Kit (#4368814, ThermoFisher Scientific) in a total volume of 50 µl, following the manufacturer’s protocol. Quantitative PCR reactions (qPCR) were performed using CFX384 Real-Time PCR Detection System (Bio-Rad). To analyze the expression of genes of interest, 2 µl of 2 × concentrated cDNA was analyzed in triplicates using inventoried TaqMan® Gene Expression Assays (list in Suppl. Table 2) and TaqMan® Universal PCR Master Mix (#4304437, ThermoFisher Scientific) in a final volume of 10 µl, according to the manufacturer’s recommendations. Cq values were obtained from CFX Manager software (version 3.1, Bio-Rad) using regression determination mode. Normalized relative expression levels were calculated using the qbase^+^ software (Biogazelle NV, Zwijnaarde, Belgium, [50], https://cellcarta.com/genomic-data-analysis/) with Bcl2l13 and Brap as reference genes for normalization. Values are given as % of Viromer + PBS control and compared with a two-way ANOVA, followed by Dunnett’s multiple comparison with Viromer control within PBS/LPS-treated groups, respectively.

#### Graphics

All figures were done in Adobe Illustrator (CS3 or CS6, Adobe Inc., CA, USA) or PowerPoint (Microsoft Office Professional Plus 2016, WA, USA).

## Results

### Characterization of Usp15 Constitutive KO and Conditional KO Mice

To analyze acute effects of Usp15 knockout after *status epilepticus* (Fig. 1b), we used a constitutive Usp15 KO mouse line (Usp15^−/−^) and respective WT littermates (Usp15^+/+^, referred to as WT in the following). To determine a possible therapeutic effect of a Usp15 knockdown in chronic epilepsy (Fig. 4a), we used a newly generated inducible Usp15 KO mouse line which allows the conditional inactivation of Usp15 via the Cre/loxP system (Usp15^fl/fl^CAGCreERT2^+^). Inducible KO mice will be referred to as Usp15^fl/fl^Cre^+^ before tamoxifen treatment and as Usp15^Δ/Δ^ after tamoxifen treatment, the respective Cre-negative littermates as Usp15^fl/fl^.

To confirm the full deletion of Usp15 in Usp15^−/−^ mice, we performed Western blot analysis of the brains at the end of the in vivo experiment (*n*_*WT,KA*_ = 8, *n*_*KO,KA*_ = 8, *n*_*WT,NaCl*_ = 7, *n*_*KO,NaCl*_ = 6; Suppl. Figure 1a). To determine if the lack of USP15 triggers a compensatory reaction, we measured protein levels of the closely related DUBs USP4 and USP11 in a subset of brains. However, protein levels of both were unchanged in Usp15^−/−^ mice compared to WT either with or without ihKA application (*n*_*WT,KA*_ = 6*, n*_*KO,KA*_ = 5, *n*_*WT,NaCl*_ = 5, *n*_*KO,NaCl*_ = 6, one-way ANOVA not significant (ns); Suppl. Figure 1b, c).

As this is the first study reporting the Usp15^fl/fl^Cre^+^/Usp15^Δ/Δ^ mouse model, we validated the protocols to achieve efficient Usp15-induced depletion: both Usp15^fl/fl^ and Usp15^fl/fl^Cre^+^ mice were treated with tamoxifen. The weight of mice during, before, and after tamoxifen treatment did not differ between genotypes (*n*_*fl/fl,KA*_ = 12, *n*_Δ*/*Δ*,KA*_ = 13, *n*_*fl/fl,NaCl*_ = 11, *n*_Δ*/*Δ*,NaCl*_ = 12, two-way ANOVA, ns, Suppl. Figure 2). In addition, upon visual inspection, behavioral parameters (feeding, grooming, social interaction) did not appear to change after tamoxifen injection. These results show that the deletion of Usp15 in adult mice does not cause overall detrimental effects. This is also an important information regarding safety considerations for potential therapeutic approaches targeting USP15 protein levels.

The efficiency of Usp15 depletion in Usp15^Δ/Δ^ mice was determined in the brains by Western blot analysis (*n*_*fl/fl,KA*_ = 8, *n*_Δ*/*Δ*,KA*_ = 9, *n*_*fl/fl,NaCl*_ = 8, *n*_Δ*/*Δ*,NaCl*_ = 11; Suppl. Figure 1d, e). USP15 protein expression levels were reduced on average to 9.3 ± 5.3% in Usp15^fl/fl^Cre + mice after tamoxifen application (i.e., Usp15^Δ/Δ^), indicating a highly efficient induced deletion (*p* < 0.001; Student’s *t* test). Again, USP4 and USP11 protein levels were unchanged after Usp15 deletion (*n*_*fl/fl,KA*_ = 6, *n*_Δ*/*Δ*,KA*_ = 6, *n*_*fl/fl,NaCl*_ = 6, *n*_Δ*/*Δ*,NaCl*_ = 6, one-way ANOVA ns; Suppl. Figure 1f, g).

To assess potential side effects due to the lack of USP15, we performed microscopic evaluation of tissues derived from multiple organs of Usp15^−/−^ in comparison with WT as well as Usp15^Δ/Δ^ mice in comparison with Usp15^fl/fl^ mice, respectively. There was no detectable difference in hematopoiesis between Usp15^−/−^ mice and WT or Usp15^Δ/Δ^ and Usp15^fl/fl^ mice, respectively. Likewise, no pathological abnormalities were observed in the spleen or bone marrow (both from sternum and femur). In the bone marrow, the cellularity was not altered, and the erythroid/myeloid ratio was maintained. None of the immune organs (mesenteric lymph node, axillary lymph node, spleen, bone marrow, Peyer’s patches, thymus, and bone marrow) showed any alteration. Any minor microscopic findings recorded were either part of the background or were attributable to tamoxifen (direct effect of tamoxifen or the injection procedure; data not shown).

### Usp15^−/−^ Mice and WT Littermates Show Comparable Severity of Behavioral Status Epilepticus

First, we tested the hypothesis whether constitutive deletion of Usp15 affects hippocampal damage and/or the inflammatory reaction early (4 days) after *status epilepticus*. At this time point, the initial cell loss following KA injection is nearly complete, and the inflammatory reaction has previously been shown to be strong in C57Bl/6 mice [51–53]. Usp15^−/−^ mice and WT littermates were injected with KA or NaCl, and the severity of the initial *status epilepticus* was monitored by video analysis. As expected, ihNaCl control mice (not video-recorded) never showed any behavioral signs resembling *status epilepticus*. Analysis of *status epilepticus* in ihKA mice using modified Racine stages (see “Methods”) revealed that the time course of the mean highest stages aligned to the time point of awakening was mostly overlapping for Usp15^−/−^ and WT mice (Fig. 1c; *n*_*WT,KA*_ = 14, *n*_*KO,KA*_ = 15). As an estimate for the intensity development of *status epilepticus*, we calculated the area under the curve per hour for the first 12 h after ihKA injection for time courses of individual animals (inset in Fig. 1c). This did not reveal any difference between WT and Usp15^−/−^ mice (multiple *t* tests with Welch’s correction ns). In addition, neither the time for each mouse to reach its individual highest stage (ihKA injection as *t* = 0 min; Fig. 1d; *t*_*WT*_ = 326.1 min, *t*_*KO*_ = 308.0 min, difference =  − 18.07 min, 95% CI [− 164,9 to 128,7], Student’s *t* test ns), nor the time to reach stages 3–6 (Student’s *t* test/Mann–Whitney test ns, Suppl. Figure 3), nor the incidence of these stages differed between Usp15^−/−^ and WT (Student’s *t* test/Mann–Whitney test ns, Suppl. Figure 3). Only mice that showed clear signs of *status epilepticus* (reaching at least stage 3) were used for further analyses. Mice were then randomly allocated to histological (*n*_*WT,KA*_ = 6, *n*_*KO,KA*_ = 6) and transcriptome analysis (*n*_*WT,KA*_ = 8, *n*_*KO,KA*_ = 9, *n*_*WT,NaCl*_ = 7, *n*_*KO,NaCl*_ = 6).

### Cell loss, Granule Cell Layer Width, and Acute Gliosis 4 Days after Status Epilepticus Does Not Differ in Usp15^−/−^ Mice and WT Littermates

**Fig. 2:**
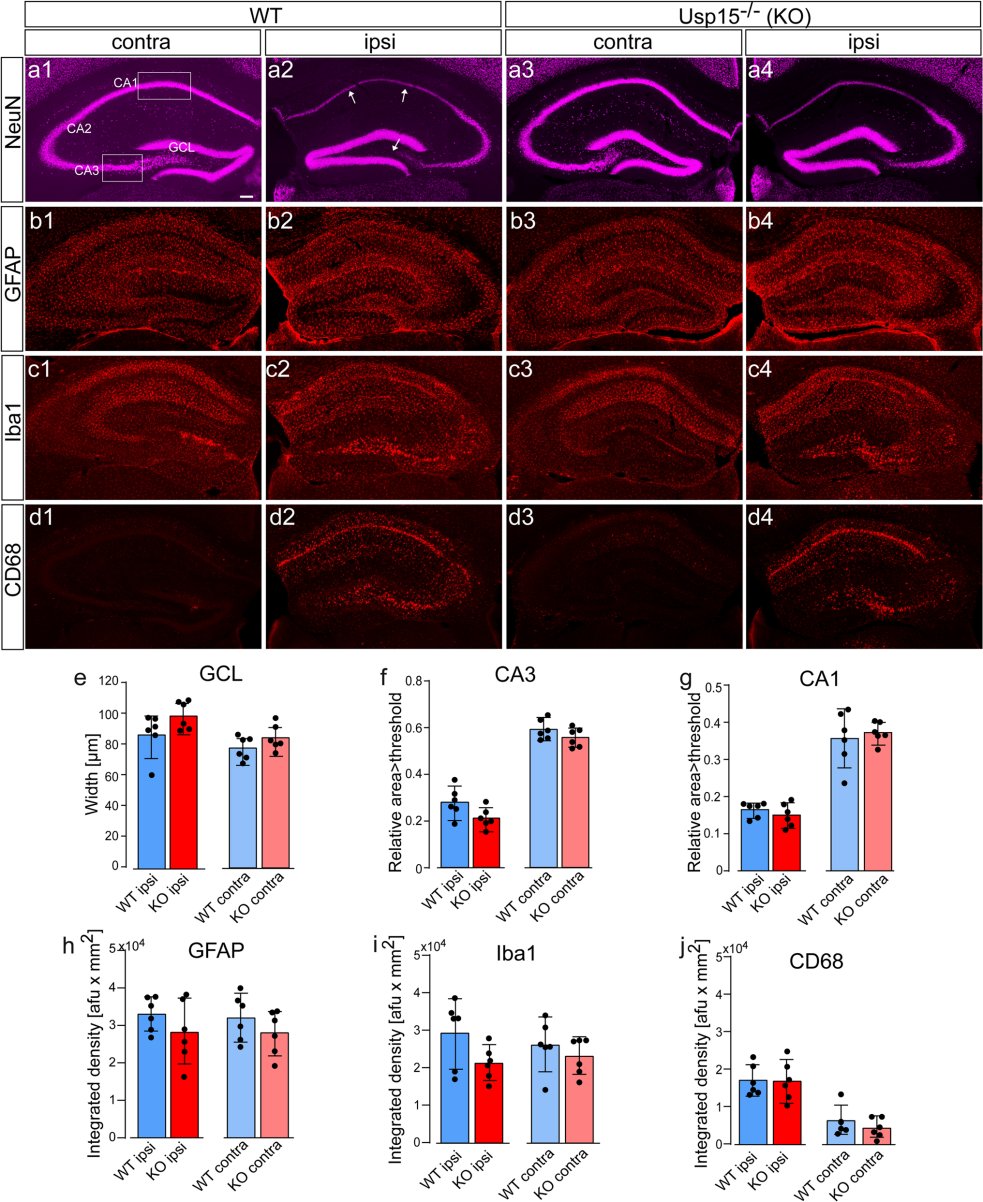
Histological hallmarks after ihKA-induced *status epilepticus* do not differ between Usp15^−/−^ mice and WT littermates.  **a1-a4** NeuN immunostaining in the ipsi- (**a1**) and contralateral hippocampus (**a2**) of wildtype (WT) and of Usp15^−/− ^(KO) mice at 4d after ihKA (**a3, a4**). Note cell loss in the hilus and CA1 (white arrows) of the ipsilateral hippocampus in both genotypes. **b1-b4** GFAP immunocytochemistry in WT and Usp15^−/−^ mice. The density and spatial distribution of astrocytes appears comparable in both genotypes. **c1-c4** Iba1 immunostaining. Microglia accumulates at the sites of strong cell loss in WT and Usp15^−/−^mice. **d1-d4** CD68 immunostaining for activated microglia does not reveal a salient difference between WT and Usp15^−/−^ mice. **e** Granule cell layer (GCL) width does not differ between genotypes for the ipsi- or contralateral hippocampus, respectively (mean ± 95% confidence intervals (CI) and individual values, Student’s *t* test). **f** CA3 NeuN density was measured in a 300*200 µm region of interest (ROI, indicated in **a1**) placed onto the pyramidal cell layer and pixels exceeding a threshold of mean grey value+1SD (measured in the whole hippocampus) were detected. Area>threshold / ROI area is given. WT and Usp15^−/−^ mice do not differ in the ipsi- or contralateral hippocampus, respectively (mean ± 95% CI and individual values, Student’s *t* test). **g** Same for CA1 but with a 400*200 µm ROI (see a1). **h** Integrated density measurement of GFAP immunostaining does not reveal differences between WT and Usp15^−/−^ mice (geometric mean ± 95% CI and individual values; unpaired Student’s *t* test after log-transformation to induce symmetry and equal variance; results were back-transformed to the original scale). **i** Same for integrated density measurement of Iba1 immunostaining. **j** Same for integrated density measurement of CD68 immunostaining. Scalebar 100µm. GCL granule cell layer, CA *cornu ammonis*

For histological analysis, we focused on the comparison of Usp15^−/−^ and WT mice, because ihKA-induced changes have already been thoroughly described [51, 54, 55]. We selected sections surrounding the injection site which show the strongest effects following the ihKA injection [53, 56]. Analysis of hippocampal NeuN immunostaining revealed neuronal loss in the hilus and a substantial thinning of principal cell layers in CA3 and in CA1. In addition, there was a beginning dispersion of the granule cell layer in the ipsilateral hippocampus. Yet, there were not any salient differences between WT and Usp15^−/−^ mice (Fig. 2a1–a4). Indeed, neither statistical comparison of granule cell layer width nor relative NeuN-positive area in the pyramidal cell layer of CA3 and CA1, which represents an indirect parameter for neurodegeneration, revealed any difference between Usp15^−/−^ and WT mice for the ipsi- or the contralateral hippocampus (Fig. 2e–g; *n*_*WT,KA*_ = 6, *n*_*KO,KA*_ = 6, unpaired Student’s *t* tests assuming equal variances for granule cell layer ns, CA3 ns, CA1 ns, respectively; see suppl. Table 3 for means and 95% CI).

To analyze the glia activation after KA injection, we performed immunostaining for GFAP, Iba1, and CD68 to label astrocytes, resident and activated microglia, respectively. At 4 days after ihKA, GFAP staining was strong throughout both hippocampi in WT and Usp15^−/−^ mice (Fig. 2b1–b4) with an accumulation of astroglia in the ipsilateral hilus and CA1 pyramidal cell layer (Fig. 2b2, b4). In the contralateral hippocampus of both genotypes, GFAP-positive cells were evenly distributed (Fig. 2b1, b3). Analysis of integrated density of fluorescence staining in both hippocampi did not reveal any significant differences (Fig. 2h, *n*_*WT,KA*_ = 6, *n*_*KO,KA*_ = 6, unpaired Student’s *t* test after log-transformation ns, respectively; see suppl. Table 3 for means and 95% CI). Iba1-positive microglia clustered at the sites of cell loss in the hilus, CA3 and CA1 pyramidal cell layer of the ipsilateral hippocampus of WT and Usp15^−/−^ mice (Fig. 2c2, c4). In the contralateral hippocampus of both genotypes, the strongest Iba1 staining was visible in dendritic layers of CA1 and in the inner molecular layer — two areas that receive commissural projections from the ipsilateral hippocampus (Fig. 2c1, c3). Quantification of integrated density of Iba1 staining in the whole hippocampus did not reveal any difference between WT and Usp15^−/−^ mice (Fig. 2i; *n*_*WT,KA*_ = 6, *n*_*KO,KA*_ = 6, unpaired Student’s *t* test after log-transformation ns, respectively; see suppl. Table 3 for means and 95% CI). Finally, the density of CD68-positive activated microglia was strongly elevated throughout the ipsilateral hippocampus following ihKA but appeared similar in WT and Usp15^−/−^ mice (Fig. 2d2, d4). The contralateral hippocampus was mostly devoid of any CD68 staining (Fig. 2d1, d3). Again, integrated density measurement did not show any difference between WT and Usp15^−/−^ mice (Fig. 2j, *n*_*WT,KA*_ = 6, *n*_*KO,KA*_ = 6, unpaired Student’s *t* test after log-transformation ns, respectively; see suppl. Table 3 for means and 95% CI).

Together, these results indicate that despite strong alterations between the contralateral and the KA-injected side in both genotypes, USP15 deficiency does not affect the pattern of granule cell dispersion, cell loss, or glia activation shortly after *status epilepticus*.

### Lack of USP15 Does Not Significantly Affect the Global Gene Expression Profile in ihKA Mice

By reanalyzing a previous dataset from the pilocarpine mTLE model [35], we identified a set of seven gene modules (modules (M) 5.o, 12.o, 16.o, 18.o, 20.o, 22.o, 24.o) which all were co-regulated in epileptic hippocampi and predicted to be regulated by the USP15-associated IFN-α/β, TGF-β, and NRF2 pathways.

**Fig. 3:**
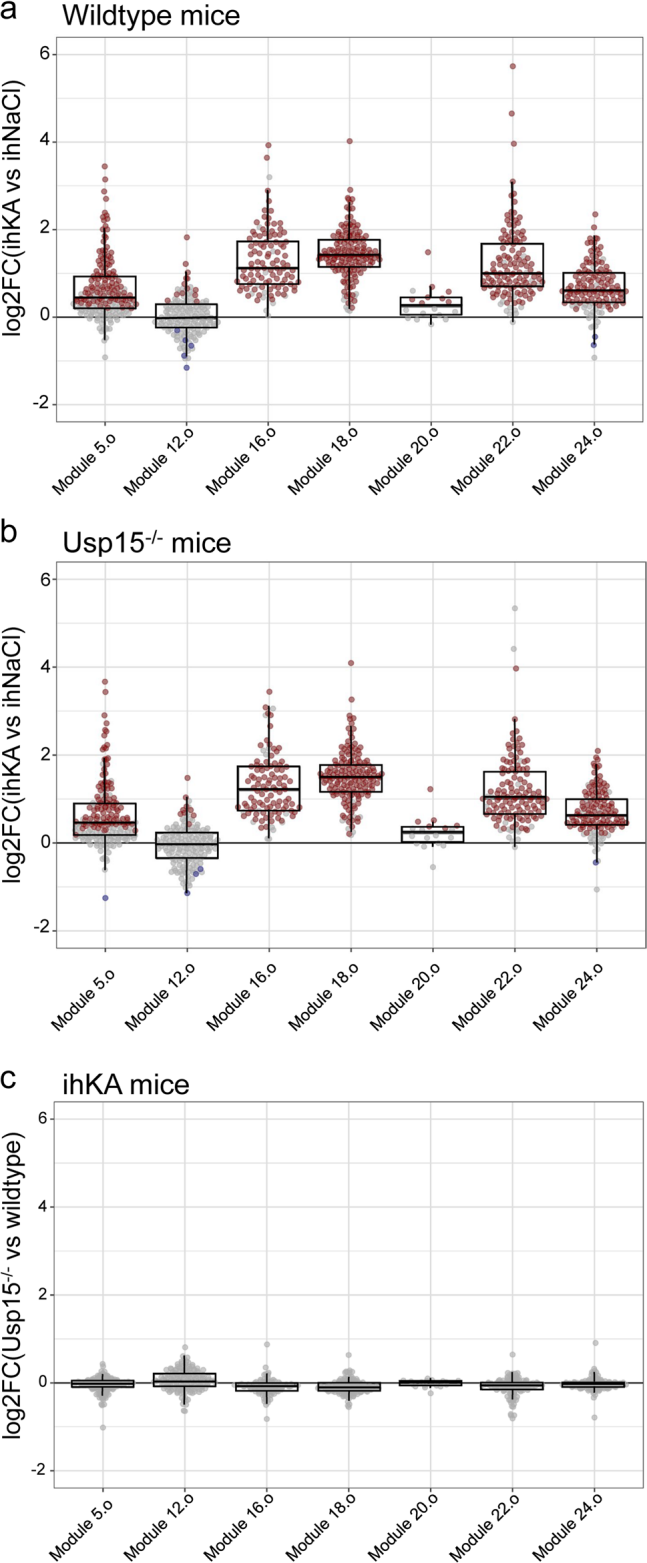
Response of the co-expression gene modules 5.o, 12.o, 16.o, 18.o, 20.o, 22.o, and 24.o predicted to be under the control of TGF-β, NRF2, and IFN-α/β pathways to ihKA and the Usp15^−/−^ genotype. Box-and-Whisker plots representing the distribution of mean log2FC per gene. Each dot represents the mean log2FC for a gene in the module; the color of the dot indicates if the gene is significantly overexpressed (red), underexpressed (blue), or not significantly differentially expressed (grey). The following comparisons were made (*n*_*WT,KA*_ = 8, *n*_*KO,KA*_ = 9, *n*_*WT,NaCl*_ = 7, *n*_*KO,NaCl*_ = 6): **a** the effect of ihKA versus ihNaCl in wild-type (WT) mice (ihKA WT vs NaCl WT: M5.o FDR < 10^−4^, M12.o FDR > 0.05, M16.o FDR < 10^−4^, M18.o FDR < 10^−4^, M20.o FDR < 0.05, M22.o FDR < 10^−4^, M24.o FDR < 10^−4^); **b** the effect of ihKA versus ihNaCl injection in Usp15^−/−^ mice (ihKA Usp15^−/−^ vs ihNaCl Usp15^−/−^: M5.o FDR < 10^−4^, M12.o FDR > 0.05, M16.o FDR < 10^−4^, M18.o FDR < 10^−4^, M20.o FDR < 0.05, M22.o FDR < 10^−4^, M24.o FDR < 10^−4^); **c** comparison of the ihKA effect in Usp15^−/−^ and WT mice (ihKA Usp15^−/−^ versus ihKA WT: M5.o FDR > 0.05, M12.o FDR < 0.05, M16.o FDR < 10^−2^, M18.o FDR < 10^−3^, M20.o FDR > 0.05, M22.o FDR < 10.^−3^, M24.o FDR > 0.05)

In the current study, the ihKA injection had a strong impact on gene expression 4 days after administration: 4791 and 2691 genes responded to KA (compared to NaCl) in the ipsi- and contralateral hippocampus of WT mice, respectively (*n*_*WT,KA*_ = 8, *n*_*WT,NaCl*_ = 7, false discovery rate (FDR) *p* ≤ 0.05). In addition, we found that the co-regulation patterns within six of the seven gene modules were conserved after ihKA-induced *status epilepticus* (suppl. Table 4) and that these six modules were significantly upregulated in response to ihKA (Fig. 3a, *n*_*WT,KA*_ = 8, *n*_*WT,NaCl*_ = 7, M5.o FDR < 10^−4^, M12.o ns, M16.o FDR < 10^−4^, M18.o FDR < 10^−4^, M20.o FDR < 0.05, M22.o FDR < 10^−4^, M24.o FDR < 10^−4^). In conclusion, while Usp15 mRNA expression was not altered itself after ihKA, gene set enrichment analysis revealed that gene modules expected to be under upstream USP15 control were conserved in the ihKA model. These data validate the use of the ihKA model to test our mechanistic hypothesis of inhibiting USP15 as a therapeutic approach to modulate pathways that play an important role in epilepsy (NRF2, IFN-α/β, and TGF-β).

Next, we assessed the impact of USP15 deficiency on the transcriptome after ihNaCl injection. Overall, the lack of USP15 only affected expression levels of three genes which were significantly differentially expressed between WT and Usp15^−/−^ mice (*n*_*WT,NaCl*_ = 7, *n*_*KO,NaCl*_ = 6, FDR *p* < 0.05): besides the downregulation of Usp15, which validated the experimental approach, we found a moderate downregulation of Rnf41 (E3 ubiquitin protein ligase) and an upregulation of Gm9770. Gene set enrichment analysis detected only mild effects of Usp15 deletion on USP15-regulated co-regulation modules. Gene modules 16.o, 18.o, and 22.o were significantly underexpressed in Usp15^−/−^ compared to WT mice after ihNaCl, but with a minimal magnitude compared to the effect caused by ihKA (data not shown).

After ihKA injection in Usp15^−/−^ mice, 4543 genes responded in the ipsilateral and 787 in the contralateral hippocampus compared to ihNaCl (*n*_*KO,KA*_ = 9, *n*_*KO,NaCl*_ = 6, FDR *p* ≤ 0.05). The same co-expression modules were upregulated in Usp15^−/−^ mice as in WT mice after ihKA (Fig. 3b, *n*_*KO,KA*_ = 9, *n*_*KO,NaCl*_ = 7, M5.o FDR < 10^−4^, M12.o ns, M16.o FDR < 10^−4^, M18.o FDR < 10^−4^, M20.o FDR < 0.05, M22.o FDR < 10^−4^, M24.o FDR < 10^−4^). When comparing the effect of ihKA on Usp15^−/−^ and on WT mice, only two genes were significantly differentially expressed (*n*_*WT,KA*_ = 8, *n*_*KO,KA*_ = 9, FDR *p* < 0.05): Usp15 was downregulated and Gm9770 was upregulated. In addition, gene modules 16.o, 18.o, and 22.o were again expressed at significantly lower levels in Usp15^−/−^ mice (as for ihNaCl), but with a minimal effect compared to the ihKA effect itself and far from any normalization of the changes brought by ihKA injection (Fig. 3c, *n*_*WT,KA*_ = 8, *n*_*KO,KA*_ = 9, M5.o ns, M12.o FDR < 0.05 (M12.o expressed at higher level), M16.o FDR < 10^−2^, M18.o FDR < 10^−3^, M20.o ns, M22.o FDR < 10^−3^, M24.o ns).

Finally, the effect of ihKA on differential gene expression in WT and Usp15^−/−^ mice was highly correlated (*r* = 0.72 in the ipsilateral hippocampus, *r* = 0.73 in the contralateral hippocampus, Pearson’s correlation; suppl. Figure 4). In conclusion, the effect of ihKA on the transcriptome was very similar in Usp15^−/−^ and in WT mice, thus ruling out the hypothesis that a constitutive deletion of Usp15 could rescue transcriptomic changes caused by ihKA-induced *status epilepticus*.

### Induced Deletion of Usp15 after Epilepsy Onset Does Not Alter Epileptic Activity in the ihKA Model of MTLE

**Fig. 4:**
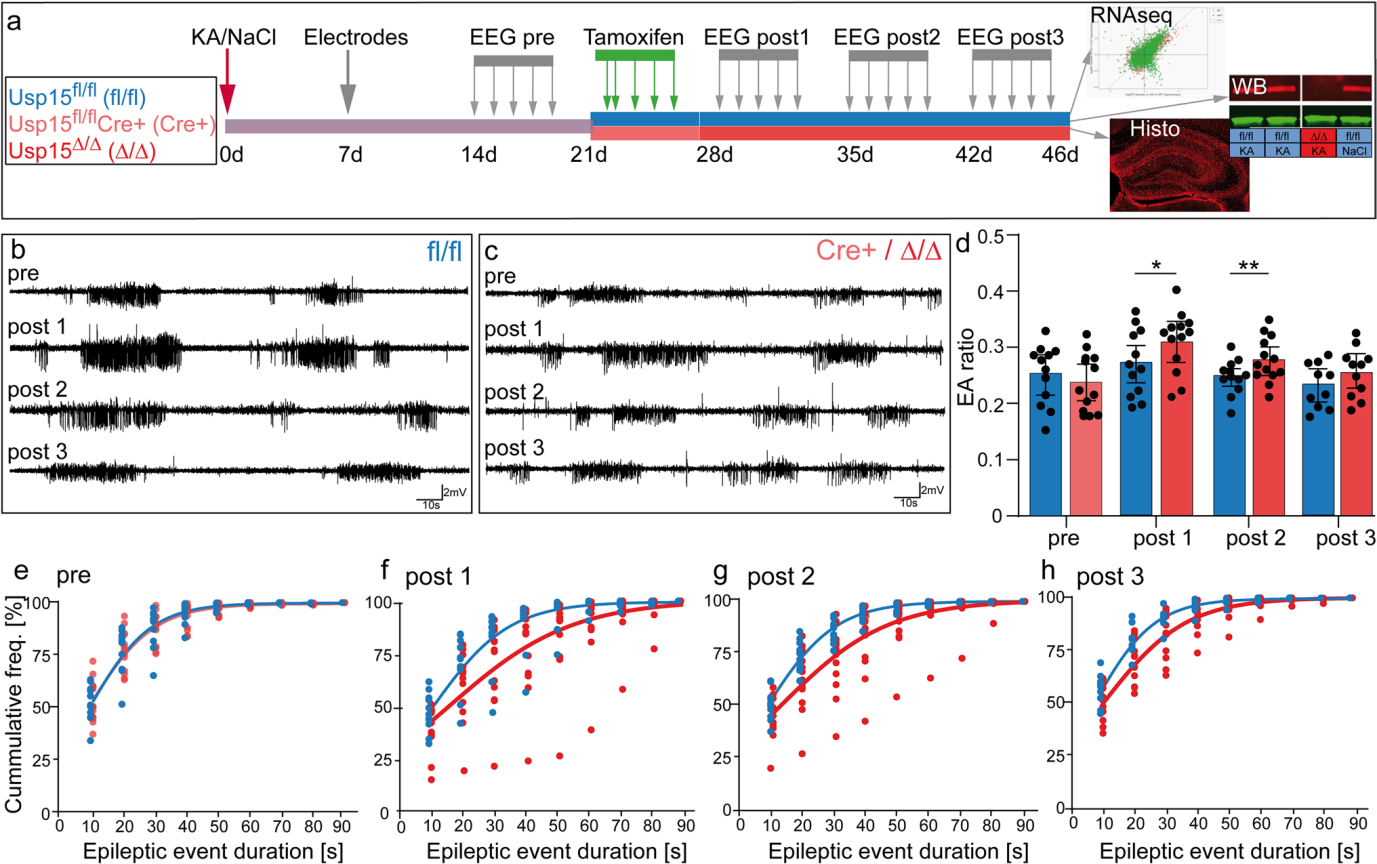
Intrahippocampal LFP recordings reveal a transient tendency for longer duration of epileptic bursts in Usp15^Δ/Δ^ mice. **a** Scheme depicting the experimental time line for the long-term experiment with Usp15^fl/fl^ and Usp15^fl/fl^Cre^+^ mice, termed Usp15^Δ/Δ^ after tamoxifen-induced Usp15 deletion. **b** Representative examples of 200-s-long cutouts from LFP recordings in a Usp15^fl/fl^ mouse. Recordings show epileptic bursts consisting of high-amplitude population spikes and intermittent fast oscillations, which can be distinguished from low-amplitude baseline. Selected traces are from the same mouse at the end of the week before tamoxifen injection (pre), and the end of weeks 1–3 (post 1–3) after tamoxifen injection and show no major changes. **c** Same as in **b** but for Usp15^Δ/Δ^ mice before (pre) and after tamoxifen-induced knockdown of Usp15. There is no salient difference between genotypes in the raw data, nor does the induced deletion of Usp15 lead to any visible changes in the raw data after tamoxifen (post versus pre). **d** Average weekly EA ratio, calculated as the time spent in epileptic bursts divided by the total recording time for Usp15^fl/fl^ (blue) and Usp15^Δ/Δ^ mice (red; means ± 95% confidence intervals and individual values). During week 1 and 2 after tamoxifen injection, the EA ratio is significantly increased in Usp15^Δ/Δ^ mice (*p < 0.05, **p < 0.01, analysis of covariance with pre-tamoxifen as the covariate). **e** Cumulative frequency of duration of epileptic bursts in 10-s bins (< 10 s, 10–20 s, etc. 80–90-s bin also contains rare events > 90 s). Individual values for each mouse are displayed, and curves represent a logistic regression analysis assuming proportional odds. Both curves overlap before tamoxifen injection. **f**, **g**, **h** Same as **e**, but for the weeks 1, 2, 3 after tamoxifen injection, respectively. **f**, **g** During week 1 and 2, the curves diverge with a right shift of the red curve, reflecting longer durations of severe epileptic bursts in Usp15^Δ/Δ^ mice compared to WT. **h** In week 3, regression curves for both genotypes converge, indicating comparable durations at the late stage. Scale bars in **b** and **c** 2 mV, 10 s

The constitutive deletion of Usp15 had no effect on strength and duration of *status epilepticus* and downstream pathophysiological processes. This might be due to compensatory processes during development. In contrast, the induced deletion of Usp15 in chronic epilepsy resembles potential therapeutic intervention and allows monitoring direct consequences. Therefore, we next tested how an induced deletion of Usp15 after epilepsy onset would alter spontaneous recurrent epileptic activity, histological hallmarks, and the transcriptome in a long-term experiment (scheme in Fig. 4a).

Starting from 2 weeks after ihKA injection, Usp15^fl/fl^Cre^+^ mice and Usp15^fl/fl^ littermates underwent LFP recordings for 5 consecutive days. This was followed by a 2-day break before tamoxifen injection and 2 (*n*_*fl/fl,KA*_ = 2, *n*_Δ*/*Δ*,KA*_ = 2) or 3 weeks (*n*_*fl/fl,KA*_ = 10, *n*_Δ*/*Δ*,KA*_ = 11) of recordings after tamoxifen (Usp15^fl/fl^Cre^+^ mice are termed Usp15^Δ/Δ^ after the tamoxifen-induced deletion of Usp15). NaCl-injected control mice (*n*_*fl/fl,NaCl*_ = 11, *n*_Δ*/*Δ*,NaCl*_ = 12) underwent the same protocol but were only occasionally recorded to confirm that these mice did not show any epileptic activity in the LFP.

Upon visual inspection, there was some inter-individual variability in epileptic activity but no salient difference between the genotypes, neither before nor after tamoxifen injection (Fig. 4b, c). After automated detection of epileptic activity [42], we determined the mean EA ratio as the cumulative duration of any kind of epileptic activity per total recording time for each week (Fig. 4d). Before tamoxifen injection, EA ratio was comparable between both genotypes, which agrees with the full expression of USP15 in both groups at this time point (Fig. 4d, means in suppl. Table 5). During week 1 and week 2 after tamoxifen injection, there was a small (~ 4%) but significant increase in EA ratio in Usp15^Δ/Δ^ compared to Usp15^fl/fl^ littermates (week 1: *n*_*fl/fl,KA*_ = 12, *n*_Δ*/*Δ*,KA*_ = 13, *p* = 0.045, week 2: *n*_*fl/fl,KA*_ = 12, *n*_Δ*/*Δ*,KA*_ = 13, *p* = 0.003, ANCOVA with pre-tamoxifen week as the covariate; suppl. Table 5), whereas in week 3 after tamoxifen, the EA ratio of both groups converged (*n*_*fl/fl,KA*_ = 10, *n*_Δ*/*Δ*,KA*_ = 11, ns).

Next, we compared the cumulative distribution of epileptic burst duration using binning with a 10-s-wide window before and after tamoxifen injection (occasional events longer than 90 s are included in the 90-s group). Individual values were fitted with logistic regression curves (Fig. 4e–h). The week before tamoxifen, regression curves were overlapping (Fig. 4e). In contrast, after tamoxifen injection, the curves for Usp15^Δ/Δ^ mice were shifted to the right for weeks 1–3, respectively, indicating longer bursts in Usp15^Δ/Δ^ mice with an odds ratio for a longer duration being ~ 1.35-fold greater than in Usp15^fl/fl^ mice for each week (Fig. 4f–h, *n*_*fl/fl,KA*_ = 12, *n*_Δ*/*Δ*,KA*_ = 13 for week 1,2, *n*_*fl/fl,KA*_ = 10, *n*_Δ*/*Δ*,KA*_ = 11 for week 3). Finally, we performed classification of epileptic bursts into different degrees of severity (severe = high spike load, moderate = medium spike load, mild = low spike load [42]). We compared the average weekly number of each class in Usp15^Δ/Δ^ and Usp15^fl/fl^ mice in weeks 1, 2, and 3 after tamoxifen using the average number before tamoxifen injection as covariate (suppl. Figure 5). This did not reveal any difference between the genotypes for severe events (ANCOVA with pre as covariate ns). For moderate and mild events, lower average numbers for Usp15^Δ/Δ^ mice were only found during week 3 and week 2, respectively (ANCOVA with pre as covariate, moderate week 3 *p* = 0.041, mild week 2 *p* = 0.047, all others ns, suppl. Figure 5).

Together, our LFP data indicate that there is a transient shift to a higher EA ratio, which is reflected in longer durations of severe events, but not in their number. Moderate and mild events also showed only minor changes. EA ratios converged during the third week after induced deletion of Usp15. In summary, the induced deletion of Usp15 did not reduce the susceptibility for epileptic activity.

After the recording phase, mice were randomly distributed to a transcriptome analysis group (*n*_*fl/fl,KA*_ = 8, *n*_Δ*/*Δ_*,*_*KA*_ = 9, *n*_*fl/fl,NaCl*_ = 8, *n*_Δ*/*Δ*,NaCl*_ = 9, one KA-injected Usp15^fl/fl^ mouse died between EEG and preparation) and a histology group (*n*_*fl/fl,KA*_ = 3 *n*_Δ*/*Δ*,KA*_ = 4, *n*_*fl/fl,NaCl*_ = 3, *n*_Δ*/*Δ*,NaCl*_ = 3).

### Histological Analysis Does Not Show Any Differences Between Usp15^∆/∆^and Usp15^fl/fl^ Mice in Chronic Epilepsy

**Fig. 5:**
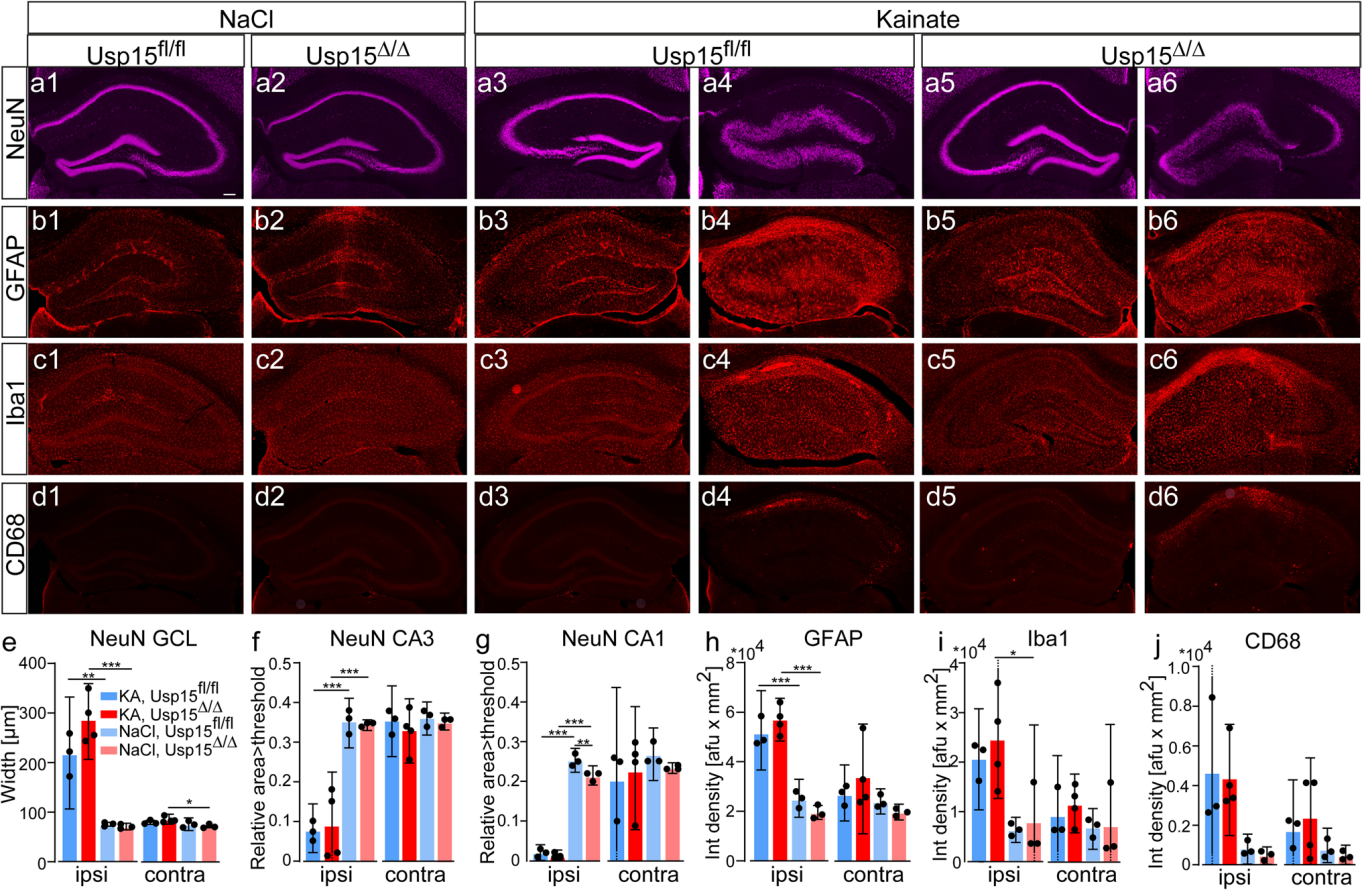
Histological analysis of Usp15^fl/fl^ and Usp15^Δ/Δ^ mice reveals differences between KA and NaCl injection, but not between genotypes. **a1-a6** Representative images of NeuN immunostaining at 6 weeks after ihKA/ihNaCl (3 weeks after tamoxifen injection) in mice that were LFP recorded. **a1, a2** NaCl-injected ipsilateral hippocampus of an Usp15^fl/fl^ and an Usp15^Δ/Δ^ mouse. **a3, a4** Contra- (**a3**) and ipsilateral hippocampus (**a4**) of a KA-injected Usp15^fl/fl^ mouse. Note the loss of pyramidal cells in CA3 and CA1 and the dispersion of the granule cell layer. **a5, a6** Same, but for an Usp15^Δ/Δ^ mouse. **b1-b6** GFAP immunostaining. In b2 the implantation track of an electrode is visible. After KA injection, GFAP-positive cells accumulate in the contralateral subgranular layer (**b3, b5**) and in the entire ipsilateral hippocampus (**b4, b6**), irrespective of the genotype. **c1-c6** Iba1 immunostaining. Iba1-positive microglia accumulate at the sites of cell death in CA3 and CA1 (**c4, c6**), irrespective of the genotype. **d1-d6** CD68 immunostaining. No activated microglia is visible in control mice (**d1, d2**) nor in the contralateral hippocampus (**d3, d5**). **d4, d6** In the ipsilateral hippocampus, CD68-positive microglia accumulated mainly in CA1. **e** Quantification of granule cell layer width for the ipsi- and contralateral hippocampus (mean, 95% confidence intervals [CI] and individual values). **f** NeuN density in CA3, area > threshold/ROI area is given (mean, 95% CI and individual values). **g** Same for CA1 indicating an almost complete loss of CA1 neurons after ihKA in both genotypes. Two-way analysis of variance (ANOVA) with Tukey multiple comparison test was performed for **e–g**. **h** Quantification of integrated density of GFAP immunostaining in the whole hippocampus (mean, 95% CI and individual values). **i** Same for Iba1 immunostaining. **j** Same for CD68 immunostaining. Two-way analysis of variance with Tukey multiple comparison test was performed on the log-transformed data for the different comparisons for **h, i**, (*p < 0.05, **p < 0.01, ***p < 0.001). Some of the 95% CI are cut for better visibility of bars. Scale bar 100 µm

To determine whether the induced deletion of Usp15 affected structural alterations after ihKA injection, we performed immunostaining for NeuN after LFP recordings, i.e., 6 weeks after ihKA/ihNacl (3 weeks after tamoxifen) (Fig. 5a1–a6). The ipsilateral hippocampus displayed strong granule cell dispersion, whereas in the contralateral hippocampus and in NaCl-injected hippocampi, the granule cell layer width appeared normal (Fig. 5a1–a6). Quantitative analysis revealed a significant increase of granule cell layer width after ihKA compared to ihNaCl injection in Usp15^Δ/Δ^ mice as well as in Usp15^fl/fl^ littermates (Fig. 5e, *n*_*fl/fl,KA*_ = 3 *n*_Δ*/*Δ*,KA*_ = 4, *n*_*fl/fl,NaCl*_ = 3, *n*_Δ*/*Δ*,NaCl*_ = 3, two-way ANOVA injection *p* < 0.0001, Tukey multiple comparison test Usp15^fl/fl^ KA-vs NaCl *p* = 0.003, Usp15^Δ/Δ^ KA vs NaCl *p* < 0.001; means and 95% CI in suppl. Table 6), but no difference between genotypes (two-way ANOVA genotype ns). In the contralateral hippocampus, a slight increase in granule cell layer width after ihKA injection was observed in Usp15^Δ/Δ^ mice only (Fig. 5e, two-way ANOVA injection *p* = 0.008, Tukey multiple comparison test Usp15^fl/fl^ KA-vs NaCl ns, Usp15^Δ/Δ^ KA vs NaCl *p* = 0.016, two-way ANOVA genotype ns).

Neuronal degeneration was indirectly determined by NeuN immunocytochemistry in CA3 and CA1. IhNaCl-treated hippocampi (Fig. 5a1, a2) and contralateral CA3 and CA1 regions after ihKA injection did not show any difference for genotype or treatment (Fig. 5a3, a5, f, g, suppl. Table 6). In contrast, the ipsilateral CA1 pyramidal cell layer was nearly gone after KA injection in both genotypes, and NeuN-positive cells in CA3 were reduced in density (Fig. 5a4, a6). The quantification of NeuN expression confirmed significantly reduced density in ipsilateral CA3 and CA1 in both genotypes in ihKA compared to ihNaCl mice (Fig. 5f, g, CA3: two-way ANOVA injection *p* < 0.0001, Tukey multiple comparison test Usp15^fl/fl^ KA-vs NaCl *p* = 0.0007, Usp15^Δ/Δ^ KA vs NaCl *p* = 0.0007; CA1: two-way ANOVA injection *p* < 0.0001, Tukey multiple comparison test Usp15^fl/fl^ KA-vs NaCl *p* < 0.001, Usp15^Δ/Δ^ KA vs NaCl *p* < 0.001; Suppl. Table 6). The comparison of genotypes, however, did not reveal any differences in CA3 after ihKA (two-way ANOVA genotype ns), but, surprisingly, in CA1, NeuN density was slightly lower in Usp15^Δ/Δ^ after ihNaCl injection (Fig. 5g, two-way ANOVA genotype *p* = 0.003, ihKA Usp15^Δ/Δ^ vs Usp15^fl/fl^ ns, ihNaCl Usp15^Δ/Δ^ vs Usp15^fl/fl^ *p* = 0.01; Suppl. Table 6). In the contralateral hippocampus, we did not observe any differences (CA3: two-way ANOVA injection and genotype ns; CA1: two-way ANOVA injection and genotype ns).

GFAP expression was weak throughout the NaCl-injected hippocampus, except for some accumulation of astrocytes along the electrode tracks (Fig. 5b1, b2). After ihKA, GFAP staining in the contralateral hippocampus was slightly increased in CA1, but a stronger increase was visible in the subgranular zone (Fig. 5b3, b5), which might reflect increased contralateral progenitor proliferation and neurogenesis after KA injection [53]. In contrast, GFAP staining was strongly enhanced throughout the ipsilateral hippocampus after ihKA, which was also seen in the quantification (Fig. 5h, two-way ANOVA injection *p* < 0.0001, Tukey multiple comparison test Usp15^fl/fl^ KA-vs NaCl *p* = 0.0003, Usp15^Δ/Δ^ KA vs NaCl *p* < 0.0001; suppl. Table 6), but again, GFAP density after ihKA injection did not differ between genotypes (Fig. 5h, two-way ANOVA genotype ns; suppl. Table 6). In the contralateral hippocampus, the comparisons did not reveal any differences for genotype or treatment (two-way ANOVA injection and genotype ns). Iba1 immunostaining was weak in NaCl-injected hippocampi as well as in contralateral hippocampi of both, Usp15^Δ/Δ^ and Usp15^fl/fl^ mice (Fig. 5c1–c3, c5). In contrast, the ipsilateral hippocampus of both genotypes showed increased Iba1 expression in the dentate gyrus and pronounced scarring in CA1 (Fig. 5c4, c6). Quantification of integrated density revealed high variability across mice, and a significant difference occurred only between ipsilateral hippocampi of KA- and NaCl-injected Usp15^Δ/Δ^ mice (Fig. 5i, two-way ANOVA injection *p* = 0.003, Tukey multiple comparison test Usp15^fl/fl^ KA-vs NaCl ns, Usp15^Δ/Δ^ KA vs NaCl *p* = 0.04, two-way ANOVA genotype ns; contralateral hippocampus two-way ANOVA injection and genotype ns; suppl. Table 6). Finally, CD68-positive activated microglia were much less prominent than shortly after *status epilepticus* and highly variable. The weak accumulation of CD68-positive cells in CA1 of the ipsilateral hippocampus after ihKA resulted in significantly higher density in group but not pairwise comparison (Fig. 5d1–d6, j, two-way ANOVA injection *p* = 0.005, Tukey multiple comparison test Usp15^fl/fl^ KA-vs NaCl ns, Usp15^Δ/Δ^ KA vs NaCl ns; Suppl. Table 6), but without a difference between genotypes in both hippocampi (two-way ANOVA genotype ns, contralateral two-way ANOVA injection and genotype ns). In summary, the treatment with KA compared to NaCl injection and the presence of epileptic activity has a strong effect on cell death and glia reaction in both genotypes, while the genotype alone plays a subordinate or no role.

### Induced Deletion of Usp15 after Epilepsy Onset Does Not Affect the Hippocampal Global Gene Expression Profile in the ihKA Model of MTLE

We investigated the gene expression profiles in the Usp15^Δ/Δ^ mice at the end of the experiment (6 weeks after ihKA/ihNacl, i.e., 3 weeks after tamoxifen). After ihKA injection, 3600 genes were differentially expressed in the ipsilateral hippocampus of Usp15^fl/fl^ mice compared to ihNaCl (1583 overexpressed/2017 underexpressed, *n*_*fl/fl,KA*_ = 8, *n*_*fl/fl,NaCl*_ = 8, FDR < 0.05), whereas only three genes were overexpressed in the contralateral hippocampus (FDR < 0.05). In Usp15^Δ/Δ^ mice, a similar number of genes was differentially expressed after ihKA (1918 overexpressed/2308 underexpressed ipsilateral; 1 overexpressed contralateral, *n*_Δ*/*Δ*,KA*_ = 9, *n*_Δ*/*Δ*,NaCl*_ = 9, FDR < 0.05). However, when comparing genotypes, we found only three genes that were significantly differentially expressed in Usp15^fl/fl^ and Usp15^Δ/Δ^ ihNaCl-injected mice (*n*_*fl/fl,NaCl*_ = 8, *n*_Δ*/*Δ*,NaCl*_ = 9, FDR < 0.05): As expected, Usp15 was underexpressed in Usp15^Δ/Δ^ mice in both hippocampi. In addition, Esr1 (estrogen receptor α, part of the inducible KO system) was found to be overexpressed in both hippocampi of Usp15^Δ/Δ^ mice, whereas dihydropyrimidine dehydrogenase (Dpyd) was slightly downregulated in the contralateral hippocampus only. After ihKA, only Usp15 and Esr1 were significantly differentially regulated in both hippocampi (*n*_*fl/fl,KA*_ = 8 *n*_Δ*/*Δ*,KA*_ = 9, FDR < 0.05). As shown above, the effect of ihKA in Usp15^Δ/Δ^ mice was highly correlated to the effect of ihKA in Usp15^fl/fl^ (suppl. Figure 4b), indicating that the ihKA response is not affected by the genotype, and Usp15^Δ/Δ^ does not influence gene expression after ihKA.

### Usp15 Knockdown Does Not Impact Microglial Functions In Vitro

Previous studies in experimental cerebral malaria and autoimmune encephalomyelitis models have implicated USP15 as a regulator of type 1 IFN responses in innate immune and T cells. It remained unclear whether the reported attenuation of CNS inflammation in Usp15 KO mice [23] is a consequence of direct action on microglia or attenuation of the peripheral innate and adaptive immune response in brain-infiltrating cells. Furthermore, there is a lack of understanding of the role of USP15 in microglial essential functions. In parallel to the in vivo target validation studies, we fill this gap by exploring the impact of Usp15 knockdown on microglial immune response and clearance functions.

**Fig. 6:**
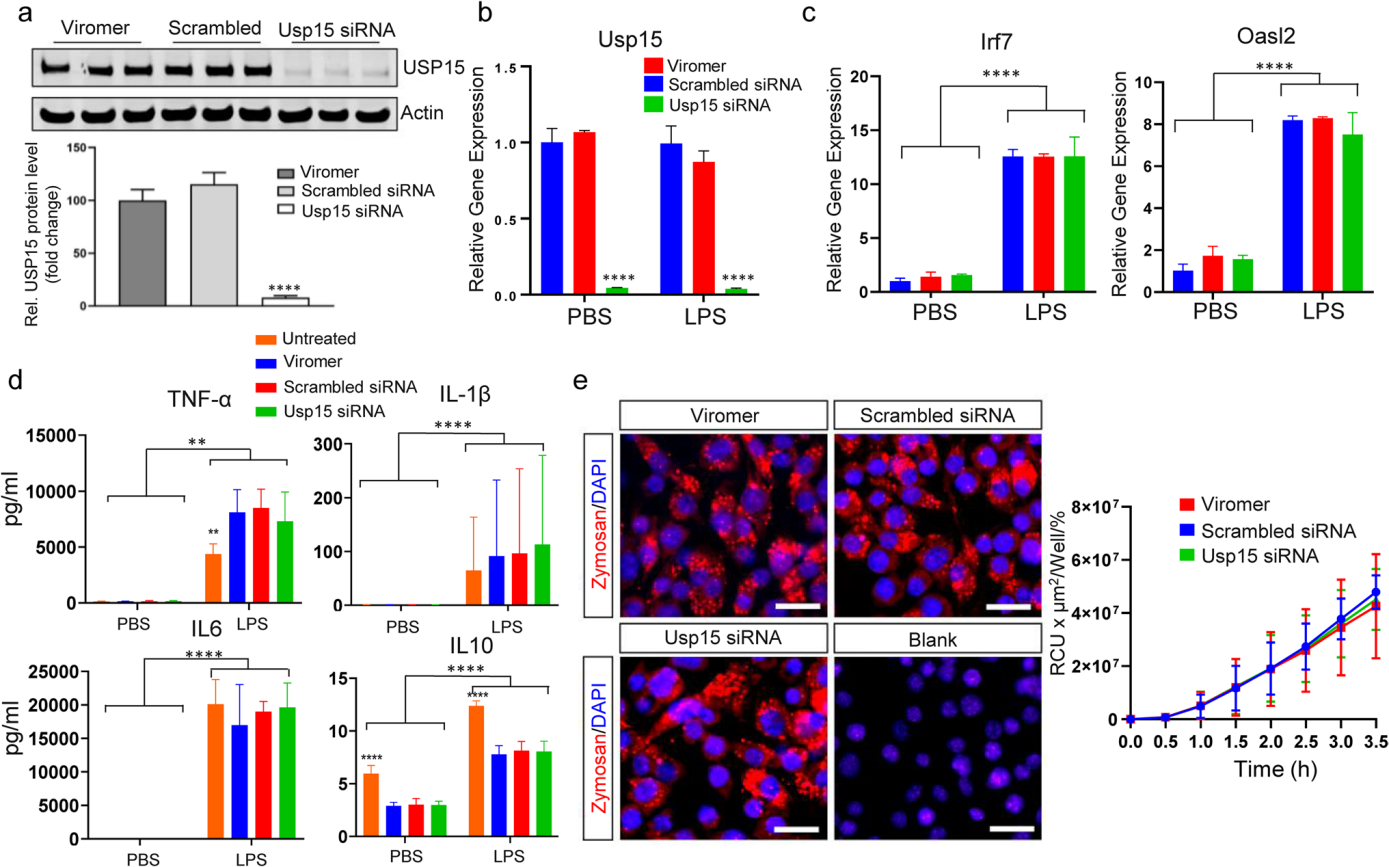
Usp15 siRNA-mediated knockdown in the BV2 cell line does not affect microglial inflammatory and clearance function. **a** USP15 protein levels at 48 h after siRNA-mediated Usp15 knockdown in unstimulated BV2 cells (Viromer and scrambled siRNA as controls). USP15 expression is significantly reduced compared to Viromer control after Usp15 siRNA application. **b** Left: Usp15 mRNA levels at 48 h after siRNA-mediated Usp15 knockdown in unstimulated BV2 cells are reduced compared to control. Right: Lipopolysaccharide (LPS) stimulation does not affect Usp15 mRNA expression. **c** SiRNA-mediated Usp15 knockdown does not affect the expression of target genes under control of USP15 (Irf7, Oasl2; see also suppl. Figure 6a) without or with LPS stimulation. **d** Usp15 knockdown does not affect the release of the cytokines TNF-α, IL-1β, IL6, and IL10 with or without LPS stimulation in BV2 cells. **e** Representative images of pHrodo®-labeled zymosan particles (red) in unstimulated BV2 microglia cells (counterstaining with DAPI in blue). Quantification of phagocytic activity does not differ following Usp15 knockdown compared to Viromer and scrambled siRNA controls. [**a**–**e** mean ± standard deviation, **a** one-way ANOVA, **b**–**d** two-way ANOVA factor 1 PBS versus LPS treatment, factor 2 siRNA treatment, with Dunnett’s multiple comparison test versus Viromer within each group, respectively, **p < 0.01, ****p < 0.0001; **e** repeated-measures two-way ANOVA.] Scale bars in **e** 25 µm

An immortalized murine microglial cell line (BV2) was used for this purpose. Prior to functional assays, efficient Usp15 knockdown using an Usp15 siRNA was assessed against a scrambled control siRNA and Viromer only. Western blot data showed > 85% decrease in USP15 protein levels at 48 h post-siRNA exposure in unstimulated BV2 cells (Fig. 6a, *n* = 3 wells each, one-way ANOVA *p* < 0.0001 with Dunnett’s multiple comparison test, Usp15 siRNA vs Viromer *p* < 0.0001, scrambled siRNA vs Viromer ns). Correspondingly, Usp15 mRNA levels were strongly reduced by siRNA-mediated knockdown in unstimulated BV2 cells (Fig. 6b; *n* = 3 wells each, two-way ANOVA factor siRNA application *p* < 0.0001, Dunnett’s multiple comparison test Usp15 siRNA versus Viromer *p* < 0.0001, scrambled siRNA vs Viromer ns).

Next, we assessed the impact of Usp15 knockdown in downstream pathway genes and cytokine release in BV2 cells challenged with 100 ng/mL LPS for 24 h. LPS stimulation did not affect Usp15 mRNA expression per se (Fig. 6b; *n* = 3 wells each, two-way ANOVA factor PBS vs LPS ns), but induced strong increases in Irf7 and Oasl2 transcript levels confirming LPS to be a relevant stimulus for the exploration of USP15 function in microglia (Fig. 6c, suppl. Figure 6; *n* = 3 wells each, two-way ANOVA, factor PBS vs LPS *p* < 0.0001). Interestingly, unlike reported previously for innate immune cells such as macrophages and neutrophils in in vivo models of cerebral malaria and autoimmune encephalomyelitis [23], Usp15 knockdown did not affect expression levels of its downstream nodes with or without LPS treatment (Irf7 and Oasl2; Fig. 6c two-way ANOVA, factor siRNA application ns; suppl. Figure 6). Next, we investigated the consequences of Usp15 knockdown for microglial inflammatory responses, more specifically cytokine release. LPS exposure of BV2 cells induced a significant release of pro-inflammatory cytokines (Fig. 6d; *n*_*untreated*_ = 3, *n*_*Viromer*_ = 9, *n*_*Scrambled*_ = 9, *n*_*Usp15siRNA*_ = 9 wells for PBS and LPS, respectively; two-way ANOVA factor PBS versus LPS for TNF-α, IL6, IL10 *p* < 0.0001, for IL-1β *p* = 0.005); however, siRNA-mediated Usp15 knockdown did not attenuate cytokine levels in LPS-stimulated microglia compared to Viromer control (two-way ANOVA factor siRNA application IL6 and IL-1β ns, IL10 *p* < 0.0001, TNF-α *p* = 0.04, Dunnett’s multiple comparison Usp15 siRNA versus Viromer all ns, scrambled RNA versus Viromer all ns, untreated vs Viromer TNF-α *p* = 0.03, IL10 *p* < 0.0001). To determine the effect of the Usp15 knockdown on microglial clearance functions, we measured phagocytic uptake of pHrodo™-conjugated zymosan particles (Fig. 6e). At 48 h following Usp15 knockdown, BV2 cells were exposed to pHrodo™-labeled zymosan particles for 2 h. Longitudinal time lapse imaging revealed that Usp15 knockdown did not affect phagocytosis (Fig. 6e; *n*_*Viromer*_ = 24, *n*_*Scrambled*_ = 24, *n*_*Usp15siRNA*_ = 24, repeated-measures two-way ANOVA ns). In addition, incubating cells with LysoTracker™ or LysoSensor™ dye did not reveal any significant alteration in lysosome acidification in BV2 cells by knockdown of Usp15 (suppl. Figure 6b, c, *n*_*Viromer*_ = 18, *n*_*Scrambled*_ = 18, *n*_*Usp15siRNA*_ = 18, *n*_*DMSO*_ = 9, *n*_*Bafilomycin*_ = 9, LysoTracker™ one-way ANOVA ns, LysoSensor™ one-way ANOVA *p* < 0.0001, Tukey’s multiple comparison test Viromer vs bafilomycin *p* < 0.0001, all others vs Viromer ns).

## Discussion

Our aim was to determine whether the upstream control of the IFN-α/β, TGF-β, and NRF2 pathways involved in neuroinflammation by blocking USP15 might serve as a treatment for pharmacoresistant epilepsy. We analyzed the hallmarks of epilepsy and neuroinflammation on the level of histology, gene expression, and epileptic seizures. In the first part of our study using constitutive Usp15 KO mice, we showed that not only *status epilepticus*, but also granule cell dispersion, cell loss, microglia, and astrocyte reaction did not differ between Usp15^−/−^ and WT mice. In addition, despite strongly altered gene expression profiles in ihKA compared to ihNaCl mice, no major differences were observed between Usp15^−/−^ and WT mice.

In the second part of our study, we showed that the conditional knockdown of Usp15 leads to a transient increase in EA ratio and duration, followed by convergence of both groups. On the histological level, granule cell dispersion, cell loss, and glia reaction were prominent in Usp15^fl/fl^ and Usp15^Δ/Δ^ mice, but did not differ between the genotypes. Finally, gene expression analysis revealed that the knockdown of Usp15 does not rescue the changes induced by ihKA injection.

As the effect of USP15 on microglia was unclear, we tested whether its knockdown affects the expression of downstream genes, cytokine release, or lysosome function using the BV2 mouse microglial cell line. The siRNA-mediated knockdown of Usp15 did not affect microglia function, neither under control conditions, nor with a pro-inflammatory challenge by applying LPS. In summary, our data indicate that the lack of USP15, despite its positive effect on other inflammatory diseases of the CNS (experimental cerebral malaria and experimental autoimmune encephalomyelitis), does not have a positive effect in mTLE.

### Status Epilepticus-Induced Changes and Alterations in Chronic Epilepsy

Our results in the ihKA model in WT littermates were in line with previous data [53, 56] indicating reproducibility of the model in the transgenic mouse line used for the current study. Our experiments showed bilateral upregulation of GFAP expression in both hippocampi at 4 days after *status epilepticus*, and a robust glial scar in the regions of major cell death on the ipsilateral side in the chronic stage (7 weeks after KA). Similarly, density of Iba1-positive microglia was strongly increased bilaterally at 4 days after *status epilepticus* with activated microglia (CD68-positive) being mostly restricted to the ipsilateral side where they might contribute to the phagocytosis of dying cells, albeit in a compromised manner as shown by earlier work [52]. At the late stage, Iba1 was still increased but only in the ipsilateral hippocampus whereas the amount of activated CD68-positive microglia was low. The spatial and temporal pattern of the glia reaction is thus in agreement with previous work [57–59]. Interestingly, the knockdown of Usp15, neither in a constitutive fashion, nor conditionally after full development of epilepsy had any major effect on this pattern.

In a previous study using pentylenetetrazole (PTZ) kindling [60], USP15 was upregulated within 24 h after the first injection with a more pronounced increase at 7 days and 30 days in the kindling protocol. While USP15 expression itself was not altered in our study (Suppl. Figure 1), gene modules regulated by the NRF2, IFN-α/β, and TGF-β pathways, expected to be under control of USP15, were overexpressed after ihKA injection. This overexpression supported the rationale of our experiment series to test whether inhibition of USP15 might serve as a beneficial therapeutic approach by modulation of these pathways.

### Possible Reasons for the Lack of an Effect of Usp15 Deletion

While we were not expecting to reduce severity of *status epilepticus* itself, we followed the hypothesis that consequential neuroinflammatory pathways could be dampened by downregulation of Usp15. This was not supported by the experiment series using Usp15^−/−^ mice. Likewise, the second experiment series did not provide a hint that deletion of Usp15 following the onset of chronic epilepsy dampens the proinflammatory response and epilepsy-related pathology.

One critical point is whether we have achieved a sufficient deletion of Usp15 in the experiments. Full absence of USP15 protein expression was confirmed in the constitutive Usp15^−/−^ mouse line, whereas in the inducible mouse line, 90% deletion efficiency was achieved. Importantly, it has to be considered that in this type of induced deletion experiments, 90% deletion does not mean that the cell still contains 10% USP15 protein but rather that 90% of the cells are lacking USP15, whereas Cre-loxP-mediated recombination failed to efficiently delete the Usp15 gene in 10% of cells. Mice with experimental cerebral malaria which carried a point mutation in the Usp15 gene in a heterozygous fashion had lower survival rates than homozygous littermates, but still performed significantly better than WT [23]. This shows that already a ~ 50% reduction of USP15 protein expression had anti-inflammatory effects in experimental cerebral malaria, indicating that a reduction to ~ 10% of the baseline level as observed in our study, should at least in theory be enough to induce clear effects.

Next, the time point of intervention might strongly affect the influence of Usp15 deletion. Previous studies [61, 62] have demonstrated that anti-inflammatory approaches can reduce downstream consequential events after *status epilepticus*. Notably, the disease-modifying potential of anti-inflammatory treatments has been demonstrated when directly administered at disease onset [12, 30] or in the chronic period [63, 64]. However, the upregulation of pro-inflammatory markers, in particular IL-1β, antagonistic suppressors of cytokine signaling and eicosanoids after ihKA-induced *status epilepticus* lasted for several hours and up to 7 days after ihKA but reached basal levels in the chronic stage [57]. When we started the deletion of Usp15 (from day 21 onwards), the inflammatory status is already established, and the impact of Usp15 deletion at this time point might be negligible. In contrast, in our study on Usp15^−/−^ mice investigating the acute effects of *status epilepticus*, the cell death and inflammatory response might have been too extensive to be impacted by USP15. This would be in agreement with previously shown overburden of microglia which are not capable of full clearance of cellular debris after ihKA [52]. Taking this into consideration, an intermediate time point for the induced deletion of Usp15 (e.g., a few days after ihKA) might be better suited to monitor consequences with regard to early-onset inflammation in future experiments. Performing longitudinal (time course) studies in the animal model might help to find optimal treatment time points and improve the outcome. However, our primary aim was to determine whether targeting USP15 might be a valid strategy for the treatment of chronic epilepsy in patients (i.e., when epilepsy is fully developed where intervention at earlier time points is not possible). Treatment during epileptogenesis is far from clinical routine given that longitudinal studies in patients which allow prediction of development of epilepsy after an initial insult are rare.

Another possible explanation might be a compensatory effect by USP4 and USP11 proteins, which carry a similar domain structure as USP15 and are highly homologous [65]. Concordantly, USP4 like USP15 was shown to strongly affect TGF-β signaling, and its inhibition reduces pathological scar formation, e.g., in skin tissue [66]. Similarly, deubiquitination of NRF2 by USP11 has been shown to stabilize NRF2 signaling in cell lines and in the context of tumors [67] and to deubiquitinase the TGF-βR1 and thereby enhance TGF-β signaling [68]. When we deleted Usp15 expression during chronic epilepsy, we observed an unexpected transient increase in EA ratio and cumulative duration of epileptic events, which may point to an involvement of compensatory mechanisms. Although we were unable to detect upregulation of USP4 and USP11 protein levels at > 3 weeks after Usp15 knockdown, these proteases might compensate the lack of USP15 function simply by enhancing enzymatic processing of the same substrates. It is conceivable that compensatory processes including a transient upregulation of USP4 or USP11 expression could have been detectable at an earlier stage, in particular at the time point when we observed increased epileptic activity (i.e., during 2 weeks after Usp15 knockdown). A compensatory overshoot of expression of the homologous DUBs might transiently increase the pathology before reaching a homeostatic stage. Finally, our in vitro study in the BV2 microglia cell line showed that USP15 or its knockdown does not have a strong effect on microglia reaction to an inflammatory challenge, as indicated by unaltered cytokine release or phagocytosis. This finding points towards a subordinate role of USP15 for the regulation of pro-inflammatory pathway in microglial cells, which has been unclear before [23]. Testing the role of USP4 and USP11 in the microglial BV2 cell line after a pro-inflammatory challenge might help to understand a possible divergence of these highly homologous DUBs in terms of their cell-type specificity.

A suitable approach to determine the interaction of the three DUBs on the systemic level in vivo and to test whether their blockade could have a beneficial effect in mTLE might be the blockade of USP15 expression in a USP4- or USP11-deficient background or a small-molecule approach simultaneously targeting the whole USP4/11/15 subfamily. Still, interpreting such studies might be very delicate, in particular since the different stages of the disease — early KA-induced cell loss, inflammation, epileptogenesis, and chronic epilepsy with recurrent seizures — might be characterized by completely different expression patterns of the individual DUBs or their combination. In addition, it is important to note that USP4, which indeed plays a role for microglial function, induced a pro-inflammatory effect instead of decreased inflammation in the spinal cord when being downregulated [69]. Consequently, a downregulation of USP4 in epilepsy might thus even have detrimental instead of beneficial effects in mTLE.

Another limitation of our study is the lumped approach, i.e., testing the expression of genes under control of the TGF-β, IFN-α/β, and NRF2 pathways in the whole hippocampus. Assuming neurons, astrocytes and microglia as the key players of epilepsy, the low effect of Usp15 knockdown in the microglial cell line does not exclude effects in the other cell types. Chen et al*.* tested the role of USP15 on glutamate-induced oxidative toxicity in a HT22 hippocampal cell line [60], i.e., investigating a neuron-specific effect. They showed upregulation of Usp15 expression in this cell line which occurred in coincidence with cell death. The knockdown of Usp15 reversed this pattern and was neuroprotective, most likely mediated by increased NRF2 and heme oxygenase-1 levels that resulted in lower production of reactive oxygen species. We might have missed such a neuroprotective effect due to the late intervention at 3 weeks after ihKA, when protection from cell death is no longer critical, as most cell death occurs within 2 days after KA injection [51]. In contrast, in the short-term experiments in the constitutive KO line, either the extreme neurotoxicity of KA might overwhelm the protective effect of Usp15 knockdown or the compensation of USP15 function by other DUBs might mask possible neuroprotective effects, as mentioned earlier. Our histological analysis of neuronal death in CA3 and CA1 might not be detailed enough to monitor more subtle changes. In addition, the effect of Usp15 knockdown in the vitality or firing properties of neurons, e.g., in acute slice studies from ihKA mice, remains to be determined.

Another factor, which is currently unclear, is the role of USP15 in astrocytes. Astrocytic changes play a major role for the development of epilepsy and the generation of epileptic seizures [70, 71], also after KA injection [72]. Despite hints towards a role of USP15 in astrocytes coming from studies in primary astrocyte cultures [23], the role of USP15 in astrocytes in vivo is still unknown, pointing towards the need for further analysis of its effect on inflammatory processes in these cells. Interestingly, microglial cells have a strong impact on the astroglial pro-epileptic changes [73]. Taking this into account — if Usp15 effects on microglial function are negligible, as suggested by our in vitro study — a possible positive effect of Usp15 knockdown in astrocytes might have been masked by the dominance of unaffected microglia. Instead of the lumped approach applied in our study, a more cell-specific analysis using single-cell RNAseq or spatial gene expression analyses would help to understand cell-type–specific effects. It is conceivable that effect of USP15 knockdown might even be divergent in the different cell types, e.g., a hypothetical protective effect in neurons might be compensated by a pro-inflammatory effect in astrocytes. This raises the need for more cell-specific analyses to better understand and eventually optimize therapeutic approaches via targeted application of Usp15 knockdown.

### Comparison to Experimental Cerebral Malaria and Autoimmune Encephalomyelitis

The rationale of our study was based on the impact seen after reduction of USP15 in the experimental cerebral malaria and autoimmune encephalomyelitis models [23]. Both diseases are associated with strong infiltration of T cells, macrophages, and neutrophils into the brain. In contrast, in ihKA-induced epilepsy, the strong immune reaction is triggered by resident microglia and astrocytes, but only to a very minor degree by invading monocytes or other invading cell types [52]. As indicated by our in vitro study, microglial cell properties seem not to be affected to a strong degree by Usp15 KO, thus partially explaining the difference to the other models. In addition, the major effect of USP15 in the two other diseases was the control of the type I IFN response. Although the IFN-α/β pathway has been shown to be upregulated in our study, genes of the IFN response (e.g., Irf7, Oasl2) were not affected by Usp15 knockdown in our microglia culture experiment. This again points to the cell-type specificity of USP15 effect and, in addition, to the interplay with other pathways. Changes in other upstream regulators of the TGF-β, IFN-α/β, and NRF2 pathways might further mask the effect of USP15 under epileptic conditions. Finally, in experimental cerebral malaria and autoimmune encephalomyelitis models, the disease develops gradually during a few days, thus allowing the optimal adaptation of compensatory activity. Instead, the injection of ihKA together with a strong *status* *epilepticus* causes a vast damage within hours, which is then followed by the (unsuccessful) attempt of brain-resident cells of the immune system to repair the massive damage. The effects of Usp15 knockdown might be too subtle to compensate for the KA-induced damage.

## Conclusions and Outlook

Overall, our study shows that the deletion of Usp15 does not have a significant impact on any transcriptional regulation in mTLE. As such, it does not invalidate the potential involvement of TGF-β, IFN-α/β and NRF2 pathways, but demonstrates that Usp15 deletion did not impact epilepsy outcomes under the conditions of our experimental setting.

A series of further experiments is conceivable to advance the understanding of the role of Usp15 in epilepsy to develop approaches for curative therapy. (I) A time-course analysis, especially during the 2 weeks after induced Usp15 knockdown, might help to understand the underlying processes, in particular why there was a transient increase of the pathology. (II) Determining the cell-type–specific effects of USP15 in microglia, astrocytes, and neurons using single-cell RNAseq might clarify the role of the individual cell types and pave the way to cell-type–specific gene therapy instead of a full knockdown. (III) After determining the role of the homologous DUBs USP4 and USP11 in the different cell types, the blockade of these homologues separately or together with USP15 in order to modify more effectively the TGF-β, NRF2, and IFN-α/β pathways involved in neuroinflammatory processes might be tested.

In addition to these, the investigation of further upstream regulators of the TGF-β, IFN-α/β, and NRF2 or other pathways involved in inflammation, specifically concerning microglia and astrocytes, is still necessary to detect possible targets for curative epilepsy treatment. Target discovery studies performed on the modules, which have been described in the pilocarpine model [35] and confirmed in the ihKA model in our study, can predict the regulatory influence of other upstream candidates. Determining the cell specificity of these candidates in cultures (in particular microglia and astrocytes) could further narrow down a set of candidate molecules to be applied in the animal model. Finally, a promising target, once found, needs to be tested in mice of both sexes as this might have different outcome and is relevant for the development of treatment of a disease with nearly equal incidence in female and male patients.

### Supplementary Information

Below is the link to the electronic supplementary material.

Supplementary file1 (PDF 632 KB)

## Data Availability

The datasets supporting the conclusions of this article are available in the article or the supplementary information. In addition, the LFP recording datasets and microscopic images are on 10.12751/g-node.3nhe4v. Transcriptome data are available on https://www.ebi.ac.uk/biostudies/arrayexpress/studies/E-MTAB-12633 and https://www.ebi.ac.uk/biostudies/arrayexpress/studies/E-MTAB-12634. For requests concerning the Usp15 mouse lines please contact K.P. Knobeloch (see corresponding authors).
