# Supplementary material for: Downregulation of Ubiquitin-Specific Protease 15 (USP15) Does Not Provide Therapeutic Benefit in Experimental Mesial Temporal Lobe Epilepsy
Source: Mol Neurobiol. 2023 Oct 24;61(4):2367–89. doi: 10.1007/s12035-023-03692-2 (PMC10973041; doi:10.1007/s12035-023-03692-2)
Supplement: Supplementary file 1 — Supplementary file1 (PDF 632 KB) [file 12035_2023_3692_MOESM1_ESM.pdf]

SUPPLEMENTARY MATERIAL

Downregulation of ubiquitin-specific protease 15 (USP15) does not provide therapeutic benefit in experimental mesial temporal lobe epilepsy

Journal: Molecular Neurobiology

Authors: Ute Häussler[1,2], João Neres[3], Catherine Vandenplas[3], Caroline Eykens[3], Irena Kadiu[3], Carolin Schramm[3], Renaud Fleurance[3], Phil Stanley[4], Patrice Godard[3], Laurane de Mot[3], Jonathan van Eyll[3], Klaus-Peter Knobeloch[5,6], Carola Haas[1,2,7,8], Stefanie Dedeurwaerdere[3]

Corresponding author:

Ute Häussler, Experimental Epilepsy Research, Department of Neurosurgery, Breisacher Strasse 64, 79016 Freiburg, Germany. Email: ute.haeussler@uniklinik-freiburg.de

Supplementary table 1 Antibodies used for Western blot

Target	Western Blot dilution	Host species	Supplier	Cat. number
**USP15**	1:1000	Mouse	Abnova	H00009958-M01
**USP11**	1:1000	Rabbit	Abcam	ab109232
**USP4**	1:1000	Rabbit	Cell Signaling	2651
**Beta-Actin**	1:10000	Mouse	Sigma	A2228

Supplementary table 2 Taqman® probes used for qPCR

Gene	Species	Assay ID (ThermoFisher)
Bcl2l13	Mouse	Mm00463355_m1
Brap	Mouse	Mm00518493_m1
Usp15	Mouse	Mm00452856_m1
Irf7	Mouse	Mm00516793_g1
Pdcd1	Mouse	Mm01285676_m1
Oasl2	Mouse	Mm01201449_m1
Hmox1	Mouse	Mm00516005_m1
Clec5a	Mouse	Mm01131767_m1
Stat3	Mouse	Mm01219775_m1
Spp1	Mouse	Mm00436767_m1

Supplementary table 3 Histological analysis of WT and Usp15$^{-/-}$ (KO) mice

Means for WT and Usp15$^{-/-}$, difference between means, 95% confidence intervals for difference and p-values of Student's t-test comparing WT and Usp15$^{-/-}$ are given. For analysis of glial markers the data have been log-transformed to achieve equal variance and geometric means are given. Afu arbitrary fluorescence units, GCL granule cell layer, int den integrated density, CI confidence interval.

	Hem	Usp15$^{-/-}$ (KO)	WT	Diff. of means/ ratio of geom. means	95% CI	p
**GCL width [µm]**	ipsi	99.54	87.2	12.34	-2.806, 27.48	0.099
	contra	84.73	78.02	6.71	- 3.561, 16.99	0.176
**Rel. NeuN^{+} area CA3**	ipsi	0.21	0.28	-0.068	-0.139, 0.003	0.057
	contra	0.56	0.59	-0.035	-0.089, 0.019	0.18
**Rel. NeuN^{+} area CA1**	ipsi	0.15	0.16	-0.014	-0.049, 0.020	0.37
	contra	0.37	0.36	0.016	-0.056, 0.088	0.63
**GFAP int den [afu/mm^2] geometric mean**	ipsi	$2.70*10^4$	$3.27*10^4$	0.825	0.597, 1.139	0.21
	contra	$2.75*10^4$	$3.15*10^4$	0.827	0.663, 1.148	0.29
**Iba1 int den [afu/mm^2] geometric mean**	ipsi	$2.09*10^4$	$2.80*10^4$	0.746	0.514, 1.082	0.11
	contra	$2.27*10^4$	$2.53*10^4$	0.898	0.636, 1.296	0.5
**CD68 int den [afu/mm^2] geometric mean**	ipsi	$1.62*10^4$	$1.68*10^4$	0.968	0.675, 1.387	0.84
	contra	$3.56*10^3$	$5.59*10^3$	0.637	0.264, 1.538	0.28

Supplementary table 4 Conservation of previously identified gene co-expression modules [1] in intrahippocampal kainate- or NaCl-injected mice

Modules predicted to be regulated by IFN-α/β, TGF-β and NRF2 pathways are identified in the "Targeted module" column. 'r^2' columns provide average squared Spearman correlation between genes belonging to each module in each condition. The significance of the r^2 (p-value and FDR [False Discovery Rate]) was assessed by performing 1000 permutations of module gene content. FDR<0.05 are underlined and displayed in bold.

**Module**	**Targeted module**	**Genes**	**r^2 (KA)**	**p-value (KA)**	**FDR (KA)**	**r^2 (NaCl)**	**p-value (NaCl)**	**FDR (NaCl)**
**MmPIL.5.o**	**Yes**	178	0.15	1.0E-03	**1.9E-03**	0.05	4.2E-01	5.9E-01
**MmPIL.16.o**	**Yes**	105	0.66	1.0E-03	**1.9E-03**	0.08	1.0E-03	**2.6E-03**
**MmPIL.18.o**	**Yes**	158	0.64	1.0E-03	**1.9E-03**	0.23	1.0E-03	**2.6E-03**
**MmPIL.22.o**	**Yes**	129	0.52	1.0E-03	**1.9E-03**	0.07	1.0E-03	**2.6E-03**
**MmPIL.24.o**	**Yes**	135	0.34	1.0E-03	**1.9E-03**	0.06	2.0E-01	3.4E-01
**MmPIL.12.o**	**Yes**	153	0.11	2.9E-02	**4.4E-02**	0.33	1.0E-03	**2.6E-03**
**MmPIL.20.o**	**Yes**	18	0.15	6.3E-02	9.3E-02	0.05	6.8E-01	8.6E-01
**MmPIL.2.o**	No	287	0.14	1.0E-03	**1.9E-03**	0.05	9.8E-01	1.0E+00
**MmPIL.24.u**	No	103	0.26	1.0E-03	**1.9E-03**	0.05	6.6E-01	8.6E-01
**MmPIL.7.u**	No	134	0.14	1.0E-03	**1.9E-03**	0.06	2.2E-01	3.5E-01
**MmPIL.9.o**	No	439	0.18	1.0E-03	**1.9E-03**	0.22	1.0E-03	**2.6E-03**
**MmPIL.17.u**	No	315	0.22	1.0E-03	**1.9E-03**	0.10	1.0E-03	**2.6E-03**
**MmPIL.14.u**	No	123	0.33	1.0E-03	**1.9E-03**	0.07	1.0E-03	**2.6E-03**
**MmPIL.29.u**	No	106	0.36	1.0E-03	**1.9E-03**	0.09	1.0E-03	**2.6E-03**
**MmPIL.21.u**	No	102	0.26	1.0E-03	**1.9E-03**	0.14	1.0E-03	**2.6E-03**
**MmPIL.20.u**	No	185	0.23	1.0E-03	**1.9E-03**	0.08	1.0E-03	**2.6E-03**
**MmPIL.6.u**	No	420	0.15	1.0E-03	**1.9E-03**	0.06	1.0E-03	**2.6E-03**
**MmPIL.27.o**	No	81	0.35	1.0E-03	**1.9E-03**	0.20	1.0E-03	**2.6E-03**
**MmPIL.28.u**	No	120	0.35	1.0E-03	**1.9E-03**	0.07	1.0E-03	**2.6E-03**
**MmPIL.22.u**	No	75	0.42	1.0E-03	**1.9E-03**	0.06	4.0E-02	8.7E-02
**MmPIL.6.o**	No	140	0.14	1.0E-03	**1.9E-03**	0.06	5.0E-03	**1.2E-02**
**MmPIL.16.u**	No	58	0.46	1.0E-03	**1.9E-03**	0.08	1.0E-03	**2.6E-03**
**MmPIL.10.u**	No	220	0.22	1.0E-03	**1.9E-03**	0.08	1.0E-03	**2.6E-03**
**MmPIL.19.u**	No	135	0.18	1.0E-03	**1.9E-03**	0.06	8.8E-02	1.7E-01
**MmPIL.23.u**	No	78	0.22	1.0E-03	**1.9E-03**	0.08	1.0E-03	**2.6E-03**
**MmPIL.5.u**	No	107	0.20	1.0E-03	**1.9E-03**	0.05	6.6E-01	8.6E-01
**MmPIL.14.o**	No	38	0.19	1.0E-03	**1.9E-03**	0.06	2.5E-01	4.0E-01
**MmPIL.10.o**	No	38	0.43	1.0E-03	**1.9E-03**	0.09	1.0E-03	**2.6E-03**
**MmPIL.18.u**	No	10	0.35	3.0E-03	**5.4E-03**	0.04	6.8E-01	8.6E-01
**MmPIL.26.u**	No	60	0.13	1.1E-02	**1.8E-02**	0.06	1.1E-01	2.0E-01
**MmPIL.17.o**	No	17	0.20	1.1E-02	**1.8E-02**	0.08	5.1E-02	1.1E-01
**MmPIL.21.o**	No	10	0.22	2.4E-02	**3.7E-02**	0.06	2.9E-01	4.4E-01
**MmPIL.2.u**	No	209	0.10	8.2E-02	1.2E-01	0.05	1.0E+00	1.0E+00
**MmPIL.25.o**	No	167	0.10	1.1E-01	1.6E-01	0.05	7.1E-01	8.7E-01
**MmPIL.15.u**	No	135	0.10	2.3E-01	3.0E-01	0.08	1.0E-03	**2.6E-03**
**MmPIL.8.u**	No	46	0.10	2.6E-01	3.4E-01	0.05	7.3E-01	8.7E-01
**MmPIL.1.u**	No	155	0.09	4.9E-01	6.1E-01	0.07	1.0E-03	**2.6E-03**
**MmPIL.11.u**	No	35	0.08	7.1E-01	8.5E-01	0.06	1.5E-01	2.7E-01
**MmPIL.13.u**	No	324	0.08	8.3E-01	9.6E-01	0.05	9.9E-01	1.0E+00
**MmPIL.15.o**	No	17	0.06	8.7E-01	9.6E-01	0.04	7.9E-01	9.1E-01
**MmPIL.13.o**	No	16	0.06	8.7E-01	9.6E-01	0.07	1.6E-01	2.7E-01
**MmPIL.11.o**	No	278	0.08	8.9E-01	9.6E-01	0.06	3.2E-02	7.3E-02
**MmPIL.8.o**	No	202	0.08	9.0E-01	9.6E-01	0.05	4.2E-01	5.9E-01
**MmPIL.4.u**	No	572	0.08	9.9E-01	1.0E+00	0.05	9.1E-01	1.0E+00
**MmPIL.26.o**	No	162	0.07	1.0E+00	1.0E+00	0.05	9.9E-01	1.0E+00
**MmPIL.4.o**	No	459	0.08	1.0E+00	1.0E+00	0.05	1.0E+00	1.0E+00

Supplementary table 5 Comparison of EA ratio in Usp15$^{fl/fl}$ and Usp15$^{\Delta/\Delta}$ mice

Values for EA ratio before tamoxifen (pre-TAM) and in weeks 1, 2 and 3 after tamoxifen injection (post) are given with difference of means and 95% confidence intervals (CI). p-values for analysis of co-variance (ANCOVA) analysis with the pre-TAM week as the covariate are given.

	Usp15$^{\Delta/\Delta}$	Usp15$^{fl/fl}$	Diff. of means	95% CI	p
**Pre TAM**	0.24	0.25	-0.01	-0.05, 0.02	
**Post week 1**	0.31	0.27	0.04	0.00, 0.09	0.045
**Post week 2**	0.28	0.25	0.03	0.01, 0.07	0.003
**Post week 3**	0.25	0.23	0.02	-0.01, 0.05	0.21

Supplementary table 6 Histological analysis of Usp15$^{fl/fl}$ and Usp15$^{\Delta/\Delta}$ mice

Means for Usp15$^{fl/fl}$ and Usp15$^{\Delta/\Delta}$ ipsi- and contralateral hippocampus, difference between means, 95% confidence intervals and p-values of analysis of variance (ANOVA) with Tukey post-hoc test comparing genotypes and treatments are given. Afu arbitrary fluorescence units, GCL granule cell layer, int den integrated density, CI confidence interval:

			Usp15$^{fl/fl}$	Usp15$^{\Delta/\Delta}$	Two-way ANOVA	95% CI of diff. Usp15$^{fl/fl}$ Usp15$^{\Delta/\Delta}$	p fl/fl vs Δ/Δ	95% CI KA-NaCl within genotype	p KA vs NaCl
**GCL width [µm]**	KA	ipsi	216.70	286.30	Injection p<0.0001	-152.4 13.40	p=0.11	fl/fl: 53.44 to 230.7	fl/fl: p=0.003
	NaCl	ipsi	74.66	69.96	Genotype p=0.13	-83.94 93.34	p=0.99	Δ/Δ: 133.4 to 299.2	Δ/Δ p<0.001
	KA	contra	78.56	84.86	Injection p=0.008	-18.35 5.754	p=0.41	fl/fl: -8.87 to 16,90	fl/fl: p=0.768
	NaCl	contra	74.55	69.83	Genotype p=0.08	-8.169 17.60	p=0.67	Δ/Δ: 2.97 to 27.08	Δ/Δ p=0.016
**Rel. NeuN area CA3**	KA	ipsi	0.08	0.09	Injection p<0.0001	-0.142 0.115	p=0.98	fl/fl: -0.41 to -0.14	fl/fl: p=0.0007
	NaCl	ipsi	0.35	0.35	Genotype p=0.91	-0.130 0.144	p=0.99	Δ/Δ: -0.39 to -0.13	Δ/Δ p=0.0007
	KA	contra	0.35	0.33	Injection p=0.51	-0.063 0.111	p=0.41	fl/fl: -0.10 to 0.09	fl/fl p=0.99
	NaCl	contra	0.36	0.35	Genotype p=0.42	-0.083 0.103	p=0.74	Δ/Δ: -0.11 to 0.07	Δ/Δ p=0.88
**Rel. NeuN CA1**	KA	ipsi	0.02	0.01	Injection p<0.0001	-0.015 0.040	p=0.52	fl/fl: -0.26 to -0.20	fl/fl p<0.001,
	NaCl	ipsi	0.25	0.21	Genotype p=0.003	0.010 0.070	p=0.01	Δ/Δ: -0.23 to -0.18	Δ/Δ p<0.001
	KA	contra	0.20	0.23	Injection p=0.40	-0.191 0.143	p=0.66	fl/fl: -0.24 to 0.11	fl/fl p=0.69
	NaCl	contra	0.27	0.23	Genotype p=0.91	-0.145 0.212	p=0.06	Δ/Δ: -0.17 to 0.16	Δ/Δ p=0.99
**GFAP int den [afu/mm^2] geometric means**	KA	ipsi	51613.11	57210.13	Injection p<0.0001	-16699.8 5505.86	p=0.43	fl/fl: 14835.5 to 38574.5	fl/fl p=0.0003
	NaCl	ipsi	24908.08	19562.65	Genotype p=0.96	-6524.05 17214.90	p=0.53	Δ/Δ: 26544.5 to 48750.3	Δ/Δ p<0.0001
	KA	contra	26911.22	34083.04	Injection p=0.09	-26812.4 12468.7	p=0.68	fl/fl: -18085.0 to 23908.2	fl/fl p=0.97
	NaCl	contra	23999.63	19749.23	Genotype p=0.75	-16746.2 25247.0	p=0.92	Δ/Δ: -53067.7 to 33974.3	Δ/Δ p=0.17
**Iba1 int den [afu/mm^2] geometric means**	KA	ipsi	20891.73	24621.50	Injection p=0.003	-19823.7 12364.2	p=0.88	fl/fl: -2911.7 to 3149.8	fl/fl p=0.11
	NaCl	ipsi	6598.27	8082.61	Genotype p=0.51	-18689.5 15720.8	p=0.99	Δ/Δ: 4449.1 to 32632.8	Δ/Δ p=0.04
	KA	contra	9463.09	11652.40	Injection p=0.24	-13928.9 9550.3	p=0.93	fl/fl: -996.7 to 15132.5	fl/fl p=0.91
	NaCl	contra	6880.77	7241.69	Genotype p=0.65	-12911.1 12189.3	p=0.99	Δ/Δ: -732.8 to 16150.3	Δ/Δ p=0.66
**CD68 int den [afu/mm^2] geometric means**	KA	ipsi	4665.97	4376.77	Injection p=0.005	-4098.70 4677.10	p=0.99	fl/fl: -885.7 to 8495.9	fl/fl p=0.12
	NaCl	ipsi	860.86	476.86	Genotype p=0.75	-4306.87 5074.86	p=0.99	Δ/Δ: -487.9 to 8287.8	Δ/Δ p=0.08
	KA	contra	1682.43	2397.45	Injection p=0.07	-3718.31 2288.26	p=0.87	fl/fl: -2276.9 to 4144.4	fl/fl p=0.80
	NaCl	contra	748.58	455.87	Genotype p=0.77	-2917.94 3503.35	p=0.99	Δ/Δ: -1061.9 to 4944.8	Δ/Δ p=0.25

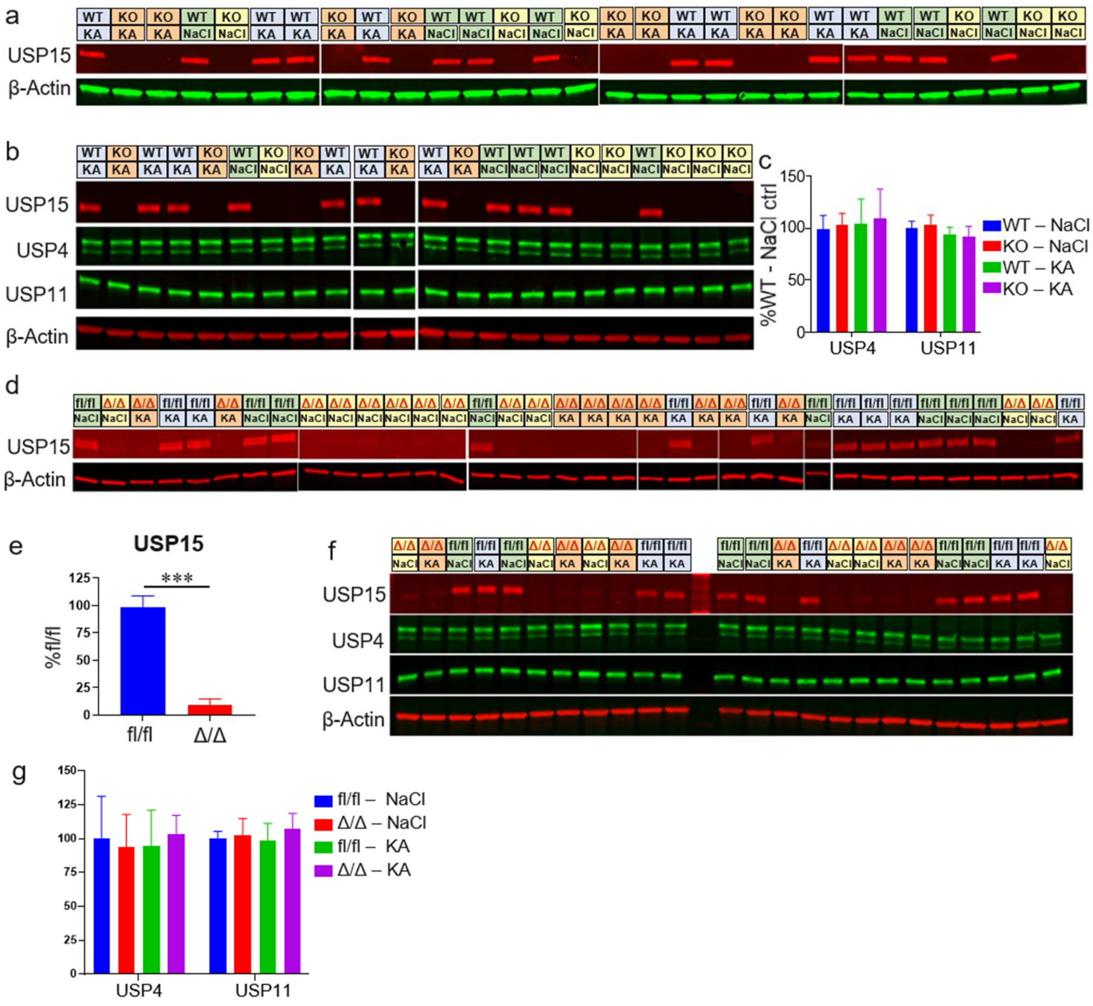

Supplementary Fig. 1 Western blot showing efficiency of Usp15 deletion and effect on protein levels of USP15, USP4 and USP11 in the brains of ihNaCl and ihKA mice

(a-c) Usp15$^{-/-}$ (KO) versus wildtype (WT) mice: (a) Western blot showing USP15 protein expression. β-Actin was used as loading control for all Western blots. (b) Western blot for USP4 and USP11. (c) Quantification of Western Blot (normalized to β-Actin) did not reveal any changes in USP4 or USP11 expression (values are given as % of NaCl-injected WT control; mean ± standard deviation (SD), one-way ANOVA). (d-g) Mice with tamoxifen-induced Usp15 deletion (Usp15$^{\Delta/\Delta}$) versus Usp15$^{fl/fl}$ mice: (d) Western blot for USP15 protein expression. (e) Respective quantification for ihKA and ihNaCl mice together (values are given as % of Usp15$^{fl/fl}$ control; mean ± SD). USP15 expression is reduced to ~10% of the control value (***p<0.001; Student’s t-test). (f) Western blot for USP4 and USP11 with the respective quantification shown in (g). The level of USP4 and USP11 expression remains unchanged under all conditions (mean ± SD; one-way ANOVA). Western blots are cropped for better visibility.

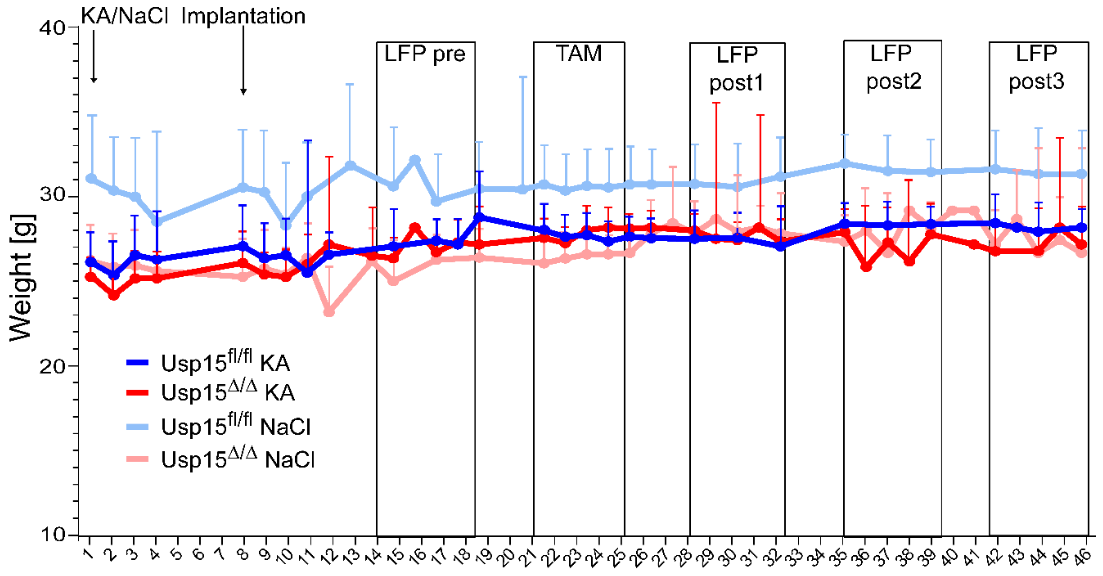

Supplementary Fig. 2 Time course of weight of Usp15$^{\Delta/\Delta}$ and Usp15$^{fl/fl}$ mice with ihKA or ihNaCl injection

Mean and 95% confidence intervals (only on top for better visibility) are displayed. There is no difference across all genotypes and treatments [(two-way factorial (genotype x injection) analysis of variance (ANOVA)]. The mean weight of ihNaCl Usp15$^{fl/fl}$ mice was higher due to some slightly older mice.

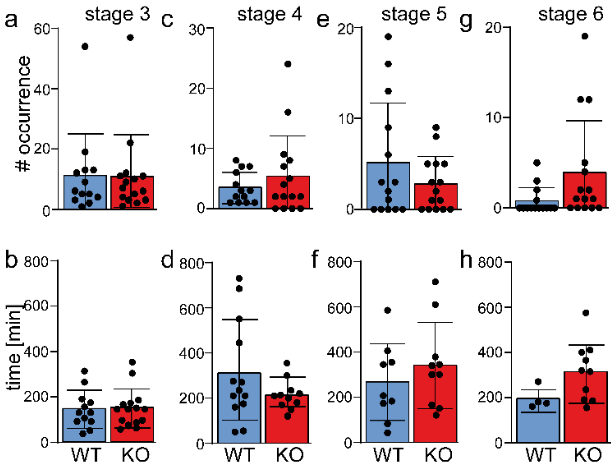

Supplementary Figure 3 Occurrence count for stages 3-6 and time to reach each stage during *status epilepticus*

(a) Occurrence count of stage 3 during at least 10h after ihKA (mean ± 95% confidence intervals, individual values) did not differ between Usp15$^{-/-}$ (KO; red) and WT (blue). (b) Time between KA injection and first stage 3 seizure (parts of this time are still under anesthesia). (c-h) Same for stage 4, 5 and 6. Note: some mice did not reach the higher stages or did not display behavior characteristic for a certain stage but instead behavior of a higher stage. There was no significant difference between Usp15$^{-/-}$ and WT for any of the stages or times (Shapiro-Wilk test for normal distribution, Student's t-test or Mann-Whitney test in case of non-normal distribution).

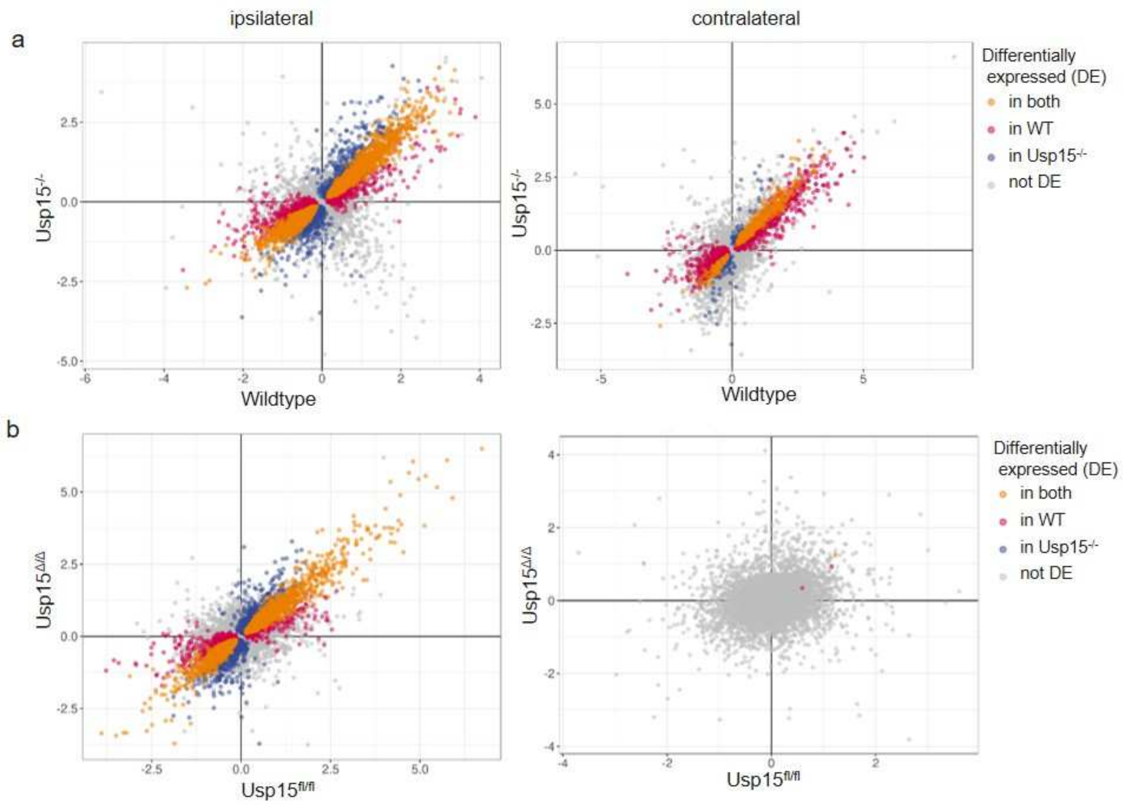

Supplementary Figure 4 Comparison of the effects of ihKA compared to ihNaCl (log2-fold change) observed in the different genotypes

a) Comparison of ihKA effect in WT and $Usp15^{-/-}$ mice in the ipsi- and contralateral hippocampus. The color of the points indicates for each gene if it is significantly differentially expressed in wildtype (WT), $Usp15^{-/-}$ mice (blue), in both (orange), or in none (grey). b) Comparison of ihKA effect in $Usp15^{fl/fl}$ and mice with induced deletion of Usp15 ($Usp15^{\Delta/\Delta}$) in the ipsi- and contralateral hippocampus. The color of the points indicates for each gene if it is significantly differentially expressed in $Usp15^{fl/fl}$ mice (red), $Usp15^{\Delta/\Delta}$ mice (blue), in both (orange), or in none (grey).

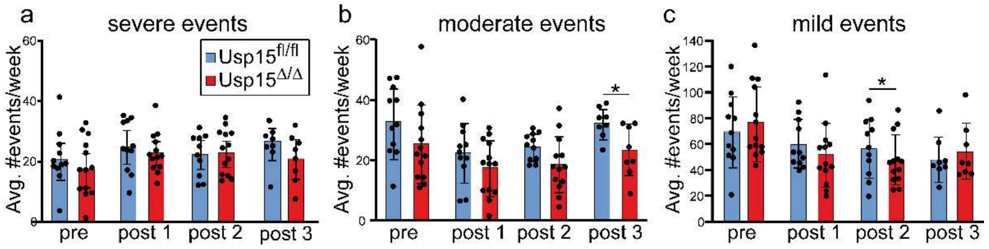

Supplementary Figure 5 Average weekly counts for epileptic bursts classified as severe, moderate or mild

(a) Weekly averages for severe bursts (i.e., bursts with high spike load) before tamoxifen (pre) and for week 1, 2 and 3 after tamoxifen for Usp15$^{fl/fl}$ (blue) and Usp15$^{fl/fl}$Cre^{+}/ Usp15$^{Δ/Δ}$ mice (red; means ± 95% confidence intervals, individual values). Comparison was made with an analysis of covariance (ANCOVA) using the average number pre-tamoxifen as baseline. There was no difference between the genotypes at any time point. (b) Same for moderate bursts (i.e., bursts with medium spike load). The average number of events was significantly lower for Usp15$^{Δ/Δ}$ mice in week 3 (*p<0.05), but comparable for all other time points. (c) Same for mild bursts (i.e., bursts with low spike load). The average number of events was significantly lower for Usp15$^{Δ/Δ}$ mice in week 2 (*p<0.05) but comparable for all other time points.

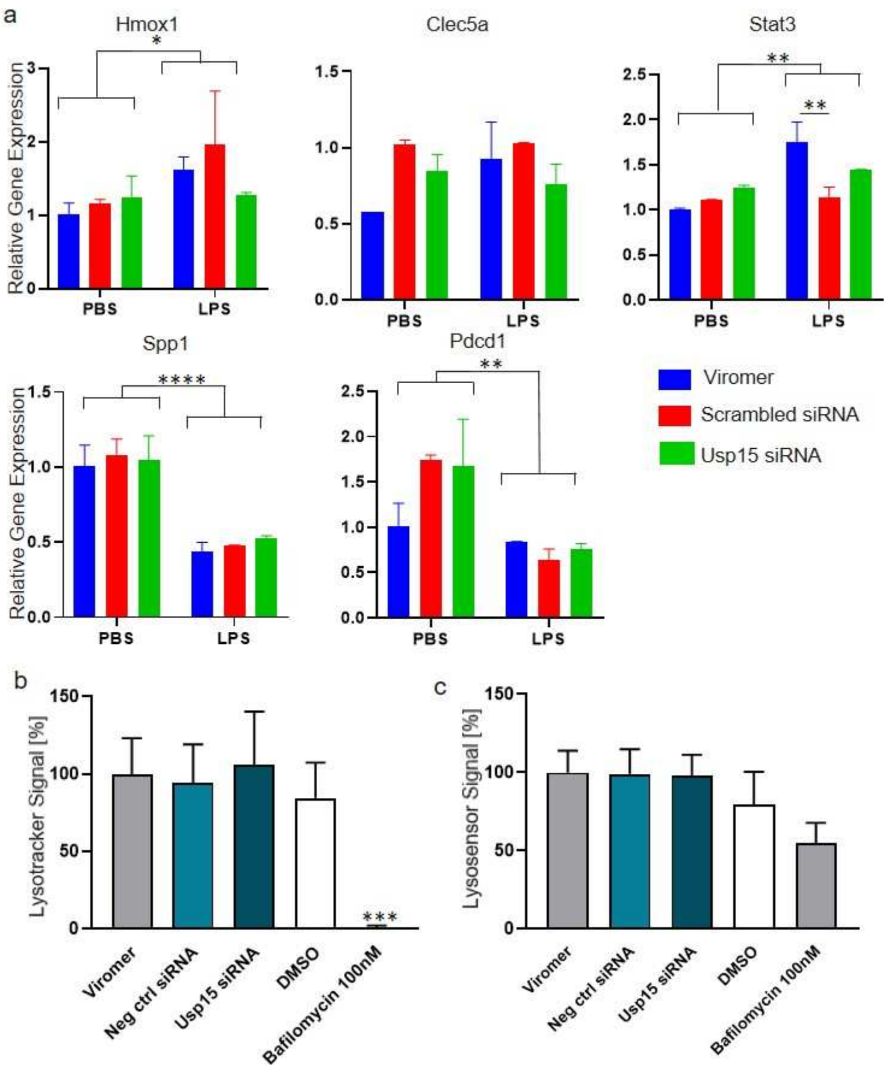

Supplementary Figure 6: SiRNA-mediated Usp15 knockdown in BV2 cell line

(A) mRNA levels (mean ± standard deviation [SD]) of genes under control of USP15 at 48h after siRNA-mediated knockdown of Usp15 in BV2 cells without and with lipopolysaccharide (LPS) stimulation. Values are given relative to Viromer + PBS control. Two-way ANOVA was performed for PBS vs LPS treatment and siRNA application, followed by Dunnett's multiple comparison test compared to Viromer within each group. LPS treatment increased the expression of inflammatory factors, but siRNA-mediated downregulation of Usp15 had not effect on the expression of Hmox1, Clec5a, Stat3, Spp1 or Pdcd1 (*$p<0.05$, **$p<0.01$, ****$p<0.0001$) (B) LysoTracker signal in BV2 cells following Usp15 knockdown is not altered at 48h post-transfection (n=1, 6-9 wells per condition, mean ± SD, Kruskal-Wallis test with Dunn's multiple comparison, ***$p<0.001$), values are given relative to Viromer control. **(C)** LysoSensor signal in BV2 cells following Usp15 knockdown is not altered at 48h post-transfection (n=1, 6-9 wells per condition, mean ± SD, Kruskal-Wallis test with Dunn's multiple comparison, ****$p<0.0001$).

References

[1] Srivastava PK, van Eyll J, Godard P, Mazzuferi M, Delahaye-Duriez A, Van Steenwinckel J, et al. A systems-level framework for drug discovery identifies Csf1R as an anti-epileptic drug target. Nat Commun. 2018 Sep 3;9(1):3561.
